# Nitrogen fertilization promoted microbial growth and N_2_O emissions by increasing the abundance of *nirS* and *nosZ* denitrifiers in semiarid maize field

**DOI:** 10.3389/fmicb.2023.1265562

**Published:** 2023-08-31

**Authors:** Setor Kwami Fudjoe, Lingling Li, Sumera Anwar, Shangli Shi, Junhong Xie, Linlin Wang, Lihua Xie, Zhou Yongjie

**Affiliations:** ^1^State Key Laboratory of Aridland Crop Science, Gansu Agricultural University, Lanzhou, China; ^2^College of Agronomy, Gansu Agricultural University, Lanzhou, China; ^3^Department of Botany, Government College Women University Faisalabad, Faisalabad, Pakistan; ^4^College of Grassland Science, Gansu Agricultural University, Lanzhou, China

**Keywords:** nitrogen fertilization, soil N_2_O emission, soil *nirS* and *nosZ* communities, potential denitrification activity, semiarid Loess Plateau

## Abstract

Nitrous oxide (N_2_O) emissions are a major source of gaseous nitrogen loss, causing environmental pollution. The low organic content in the Loess Plateau region, coupled with the high fertilizer demand of maize, further exacerbates these N losses. N fertilizers play a primary role in N_2_O emissions by influencing soil denitrifying bacteria, however, the underlying microbial mechanisms that contribute to N_2_O emissions have not been fully explored. Therefore, the research aimed to gain insights into the intricate relationships between N fertilization, soil denitrification, N_2_O emissions, potential denitrification activity (PDA), and maize nitrogen use efficiency (NUE) in semi-arid regions. Four nitrogen (N) fertilizer rates, namely N0, N1, N2, and N3 (representing 0, 100, 200, and 300 kg ha^−1^ yr.^−1^, respectively) were applied to maize field. The cumulative N_2_O emissions were 32 and 33% higher under N2 and 37 and 39% higher under N3 in the 2020 and 2021, respectively, than the N0 treatment. N fertilization rates impacted the abundance, composition, and network of soil denitrifying communities (*nirS* and *nosZ*) in the bulk and rhizosphere soil. Additionally, within the *nirS* community, the genera *Cupriavidus* and *Rhodanobacter* were associated with N_2_O emissions. Conversely, in the *nosZ* denitrifier, the genera *Azospirillum*, *Mesorhizobium*, and *Microvirga* in the bulk and rhizosphere soil reduced N_2_O emissions. Further analysis using both random forest and structural equation model (SEM) revealed that specific soil properties (pH, NO_3_^−^-N, SOC, SWC, and DON), and the presence of *nirS*-harboring denitrification, were positively associated with PDA activities, respectively, and exhibited a significant association to N_2_O emissions and PDA activities but expressed a negative effect on maize NUE. However, *nosZ*-harboring denitrification showed an opposite trend, suggesting different effects on these variables. Our findings suggest that N fertilization promoted microbial growth and N_2_O emissions by increasing the abundance of *nirS* and *nosZ* denitrifiers and altering the composition of their communities. This study provides new insights into the relationships among soil microbiome, maize productivity, NUE, and soil N_2_O emissions in semi-arid regions.

## Introduction

Nitrogen (N) fertilizer plays a crucial role in plant nutrition, and its insufficiency can hinder crop productivity (Gitelson et al., 2003; Berthrong et al., 2014; Thomas et al., 2015). The provision of adequate N fertilizer supply is essential for, enhancing crop biomass and grain yields, as well as influencing the structure of microbial communities such as denitrifying bacteria (Fan et al., 2005; Dai et al., 2015). China accounts for approximately 39% of global N fertilizer consumption as the largest chemical N fertilizer user according to IFA (2021). The utilization of N fertilizer has remarkably contributed to the rapid growth in maize production and has had a positive impact on food security (Wu and Ma, 2015; Liu et al., 2018a; Shah and Wu, 2019). Additionally, excessive application of N fertilizer can lead to low NUE and pose severe environmental risks, including soil degradation, nitrate leaching, ammonia (NH_3_) volatilization, and emissions of N_2_O (Snyder et al., 2009; Liang et al., 2019; Guo et al., 2021).

N_2_O emission has emerged as a potent greenhouse gas that has garnered increasing attention due to their significant role in global warming and ozone depletion. N_2_O emission has gained recognition as one of the major contributors to climate change and possesses a higher impact per unit of emissions compared to carbon dioxide (CO_2_) (Cuello et al., 2015; Schmidt et al., 2019). Among the various sources of N_2_O emissions, agriculture stands out as the largest contributor to anthropogenic N_2_O emissions, primarily due to synthetic fertilizers and livestock manure (Yang et al., 2021a). When nitrogen-based fertilizers are applied to crops, they have the potential to undergo nitrification and denitrification processes, resulting in the emission of N_2_O into the atmosphere. However, studies have demonstrated that enhancing NUE can significantly mitigate N_2_O emissions from agricultural systems, reducing them by as much as 60% while contributing to climate change mitigation (Liu et al., 2018a). Moreover, besides the benefits of climate change mitigation, improving NUE can lead to increased crop yields, reduced fertilizer expenses, and enhanced soil health (Fan et al., 2005; Huang et al., 2020a).

Soil microbial denitrification is an aerobic process that involves the sequential reduction of nitrate (NO_3_^−^) to nitrite (NO_2_^−^), nitric oxide (NO), nitrous oxide (N_2_O), and finally to dinitrogen gas (N_2_). Specific enzymes facilitate this process and encompass transcriptional and nitrogen-fixing activities (Tao et al., 2018; Huang et al., 2019). Key genes, namely *nirK*, *nirS*, and *nosZ*, play a crucial role in converting N_2_O to nitrogen gas (N_2_) and are significant regulators of N_2_O emissions. The *nirK* and *nirS* genes are responsible for converting N_2_O to nitric oxide (NO), while the *nosZ* gene is responsible for reducing N_2_O to N_2_ (Shi et al., 2019; He et al., 2020). While N fertilization impacts the diversity and structure of *nirS* and *nosZ* communities, limited research has been conducted on the effects of long-term inorganic N fertilization on denitrification in soils of semiarid regions (Schmidt et al., 2019; Ullah et al., 2020). Changes in soil pH, soil water content, temperature, soil organic carbon (SOC), total nitrogen (TN), and nitrate-nitrogen (NO_3_^−^-N) can exert an influence on the composition and structure of *nirS*-, and *nosZ*-harboring denitrifiers. Consequently, these changes, in turn, have the potential to affect the rate and pathways of N_2_O production, specifically the activities of nitrification and denitrification (Han et al., 2020). The emission of N_2_O is significantly influenced by long-term N fertilization and soil properties, as they impact various factors such as nutrient transport, microbial functions, soil composition, and soil PDA (Ouyang et al., 2018; Pang et al., 2019; Huang et al., 2020b). More studies are needed to better understand the specific effects of N fertilization on denitrification processes in these environments.

Maize crop is extensively grown in various regions worldwide, including semiarid areas like the Loess Plateau, which holds significant agricultural importance. The semi-arid Loess Plateau (SALP) in northwestern China is recognized as one of the most fragile agroecosystems globally, heavily reliant on limited and unpredictable rainfall (Qiu et al., 2014; Zhang et al., 2018; Fudjoe et al., 2021). To address this challenge, farmers frequently adopt the use of plastic mulch to in reduce water evaporation and retain moisture, fulfilling the crop’s essential growth requirements and resulting in enhanced crop yields and improved water use efficiency in the bulk and rhizosphere soil (Bu et al., 2014; Lamptey et al., 2019; Zhang et al., 2021). The bulk soil and rhizosphere soil play distinctive roles in influencing soil microbial communities. Soil microbial communities are shaped by various factors, with the rhizosphere playing a crucial role in impacting crop growth and soil fertility (Wen et al., 2017; Amadou et al., 2020). The bulk soil, which is not penetrated by plant roots, typically contains lower levels of natural organic compounds and microbiome exudates, which facilitate symbiotic associations and nutrient cycling compared to the rhizosphere soil (Liang et al., 2019; Schmidt et al., 2019).

The complex soil population consists of diverse microbial communities that play a vital role in the dynamic global ecology (Mamet et al., 2019). Hence, employing a network-based approach proves valuable in examining the connections between microbial communities and ecological factors, thereby identifying potential keystone species that contribute significantly to carbon and nitrogen cycling (Barberán et al., 2012; Fudjoe et al., 2022). Keystone taxa are essential for maintaining coherence and enhancing the performance of microbial communities (Williams et al., 2014; Chen et al., 2019a). These taxa species possess closely associated functional traits that significantly influence the network structure, thereby altering soil microbial communities involved in N_2_O emission. Although recent studies have explored the correlation networks of denitrification in natural forest soil and arable black soil (Herren and McMahon, 2018; Chen et al., 2019b; Han et al., 2020), limited knowledge exists regarding the impact of nitrogen fertilization rate treatments on microbial networks in the semi-arid Loess Plateau.

Gaining insights into the factors influencing changes in soil denitrification communities and their impact on N_2_O emissions is important for achieving sustainable agriculture in the semi-arid Loess Plateau (Lamptey et al., 2019; Ullah et al., 2020). In this study, we propose that different rates of N fertilizer application would alter N_2_O emission, PDA, soil physicochemical properties, abundance and diversity of denitrifying communities, and maize NUE. The objectives of our experiment were as follows: (i) to investigate the effects of varying N fertilizer rates on the abundance, diversity, and Composition of *nirS*- and *nosZ*-harboring communities in the bulk and rhizosphere soil; (ii) to evaluate the influence of different N fertilizer rates on N_2_O emissions, PDA, maize yield, and NUE; and (iii) to examine the associations and mechanisms linking N_2_O emissions, soil physicochemical parameters, PDA, maize productivity, and NUE. This research aims to enhance our understanding of selected soil factors driving the composition of *nirS*- and *nosZ*-harboring communities and maize NUE in semiarid soils for agricultural production in the Loess Plateau region.

## Materials and methods

### Overview of the experimental site

From 2012 to 2021, we conducted various fertilizer treatments on a maize field located at Gansu Agricultural University in Gansu Province, NW China (35°28′N, 104°44′E). Within the 2021 cropping season, soil samples was collected for microbial data analysis and gathered data related to maize crops. The study site is characterized by a semi-arid climate on the Loess Plateau, with 140 frost-free days annually and an elevation of 1971 meters above sea level. The region experiences an average annual precipitation of 400 mm, a mean annual temperature of 10.8°C, an evaporation rate of 1,531 mm, and an average annual radiation of 5,930 MJ m^−2^. The soil in this area originates from aeolian deposits, containing at least 50% sand and classified as Calcaric Cambisol according to FAO (1990) guidelines. Additional physiochemical properties of the soil before the start of the experiment can be found in Supplementary Table S1.

### Design of the experiment and sample collection

The experiment utilized a randomized complete block design with four treatments and three replicates per treatment. The nitrogen fertilizer fertilizers rates were 100 (N1), 200 (N2), and 300 (N3) kg N ha^−1^, while no nitrogen was applied for control (N0). Each treatment was replicated three times. All plots received an application of 150 kg P_2_O_5_ ha^−1^ at the time of application of first nitrogen dose. Due to the high potassium (K) content (220 mg kg^−1^) in the soil of the region, no additional K fertilizer was applied, as it was deemed sufficient for crop growth (Yue et al., 2022). Nitrogen were supplied as urea (16% N) and triple superphosphate (16% P_2_O_5_) respectively. The experiment comprised 16 plots, each measuring 3 m × 14.2 m in area. Nitrogen fertilizer was applied in two stages: one-third of the total nitrogen fertilizer was evenly spread on the soil surface before maize sowing, while the remaining two-thirds were applied to the soil at the six-leaf stage of the maize. Prior to sowing, nitrogen (N), and phosphorus (P) fertilizers were hand-applied via broadcasting and incorporated by shallow cultivation, followed by harrowing. However, during the six-leaf stage of maize growth, N application was carried out using a handheld injection device positioned alongside each row of maize plants due to the presence of plastic mulch.

In late April, maize seeds of the Pioneer 335 cultivar were planted at a density of 52,500 plants per hectare. Maize Pioneer 335 cultivar are disease and pest resistant. It is also estimated to mature within a time frame of 5 to 6 months with an estimated grain yield of 34,382 kg ha^−1^. The field was prepared with wide (0.7 m) and narrow (0.4 m) ridges covered with transparent plastic film. The transparent plastic mulch (polyethylene film) was 1.01 mils thick, 4 feet wide, in rolls 2,000 feet long. It was available in a clear (transparent) color. Holes were created in the film over the furrows to facilitate the collection of precipitation. The seeds were sown within the furrows (Supplementary Figure S1). After placing the film over the soil, it was perforated using a handheld device (Lamptey et al., 2019). Manual weeding (hand) was performed to control weeds throughout the growing season. The maize grain was harvested in late September.

### Soil collection and evaluation

During the flowering stage of maize, soil samples were collected at a depth of 20 cm from the field in the 2021 cropping season using a 5 cm diameter auger. Randomly selected *bulk* soil samples were collected from various locations within the field, while *rhizosphere* soil samples were obtained by sampling the soil adhered to the crown root. A total of 12 soil samples (three replicates for each of the four treatments) were collected. To create a uniform sample, 10 soil cores were combined from each plot. The samples underwent processing to remove stones and surface debris and were then sieved using a 2 mm sieve mesh. To prevent cross-contamination, the auger and sieve mesh were cleaned with ethanol and clean tissue paper before collecting new samples. The soil samples were promptly placed on dry ice for transportation to the laboratory. Half of each sample was air-dried for subsequent chemical analysis measurements, while the other half was stored at −80°C for microbial community analysis.

To analyze the soil pH, a mixture of deionized water and soil was prepared in a ratio of 1:2.5 (mass to volume). The pH of the resulting extract was measured using a pH meter (Mettler Toledo FE20, Shanghai, China) (Amadou et al., 2020). Total nitrogen (TN) content was determined using the Kjeldahl method, while the organic carbon content (SOC) was measured using the Walkley-Black wet oxidation method (Bao, 2000). The concentration of dissolved organic nitrogen (DON) was quantified using the multi-N/C 2100 s Analyzer (Analytik Jena, Germany). Ammonium nitrogen (NH_4_^+^-N) and nitrate nitrogen (NO_3_^−^-N) concentrations were determined using a spectrophotometer at a wavelength of 550 nm and 204 nm, respectively, (UV-1800, Mapada instruments, Shanghai, China) after extracting the soil with 2 M KCl (Bremner, 1996). The available phosphorus (AP) concentration was determined via the molybdenum-blue method after extracting the soil with sodium bicarbonate (Olsen et al., 1954). Soil water content (SWC) was assessed by subjecting the soil to oven-drying at 105°C for 24 h (Fudjoe et al., 2022).

### Soil measurement of potential denitrification activity and yield

The soil’s potential denitrification activity (PDA) was assessed during the maize cropping season of in 2021 using a modified version of the acetylene inhibition method (Philippot et al., 2011). In this approach, approximately 4 grams each of soil (equivalent to the dry weight) from a total of 12 soil samples (four treatments with three replicates) were incubated in an incubator with a solution consisting of KNO_3_ (50 μg NO_3_^−^-N g^−1^ dry soil), glucose (0.5 mg C g^−1^ dry soil), and sodium glutamate (0.5 mg C g^−1^ dry soil) in a 150 mL sterile flask. The mixture was gently mixed and then incubated at 28°C in an incubator. The atmosphere within each sterile flask was evacuated to establish anaerobic conditions and inhibit N_2_O-reductase activity, and purged with a 90:10 He-C_2_H_2_ gas combination. Gas samples were collected at the start of the incubation after 2 h and analyzed for N_2_O concentrations using a gas chromatograph (Agilent, 7890A, United States) equipped with an electron capture detector. The PDA value was determined as ng N-N_2_O produced per hour per gram of dry soil.

The aboveground dry biomass was assessed by subjecting it to oven drying until a constant weight was achieved during the maize cropping seasons of 2020 and 2021. The grain yield was measured after harvesting and air-drying all maize cobs obtained from the plot. The nitrogen use efficiency (NUE) was calculated by subtracting the nitrogen uptake in the treatment without nitrogen fertilizer from the nitrogen uptake in the treatment with nitrogen fertilizer and then dividing this difference by the nitrogen application rate (Liu et al., 2018b).

## Collection and analysis of N_2_O emission samples

Gas samples were collected via the static chamber technique, and the concentration of nitrous oxide (N_2_O) was determined using a gas chromatography instrument (Agilent 7080B, Santa Clara, United States) at regular intervals (monthly or bi-monthly) throughout the maize growing seasons of 2020 and 2021. A total of 12 soil samples (three replicates for each of the four treatments) were collected. To minimize the impact of thermal heat during gas sampling, the sealed containers were constructed with an opaque outer lid covered with crenelated container foil. The dimensions of each container were 0.38 m × 0.35 m × 0.36 m. Additionally, two fans were installed inside the container to ensure appropriate gas circulation before sampling. To reduce the influence of daily temperature variations, N_2_O gas samples were collected within a particular time frame (between 9:00 and 11:00) during varied sampling periods (0, 10, and 20 min once the chamber was closed) via a 60 mL plastic gas-tight syringe. After collection, the gas samples were preserved in airtight aluminum bags (manufactured by Dalian Delin gas packing, China). Gas chromatography (Agilent 7890A, United States) was equipped with an electron capture detector for analyzing the gas samples.

(1) The N_2_O fluxes (NF, mg m^−2^ h^−1^) were determined by applying Eq. (1) following the methodology outlined in the study by Huang et al. (2019);


(1) $$\mathrm{NF}=\frac{273}{273+T}\times \frac{44}{22.4}\times 60\times 10^{-3}\times h\times \frac{\mathrm{dc}}{\mathrm{dt}}$$

The equation involves various parameters: T (°C) represents the air temperature, 44 is the molecular weight of N_2_O, 22.4 (L mol^−1^) is the molecular volume at 101 kPa, 60 × 10^−3^ is an alteration factor, *h* stands for the height of the chamber, and dc/dt denotes the rate of change in N_2_O concentration (c) over time (t).

(2) N_2_O cumulative emissions (NE, Kg ha^−1^) were calculated using Eq. (2) based on Tao et al. (2018);


(2) $$\mathrm{NE}=\sum \left[\frac{{\mathrm{NF}}_{i+1}+{\mathrm{NF}}_{i}}{2}\times \left(t_{i+1}-t_{i}\right)\times 24\times 10^{-2}\right]$$

Where *i* + 1 and *i* are the last and current measurement dates, respectively, and *t* is the temperature on the number of days after sowing.

### Soil microbial DNA extraction and illumina processing of denitrification community

Analyses of functional genes (*nirS* and *nosZ*) were done with clean chimera tags from the 12 samples (four treatments × three replications). To obtain the total genomic DNA from the soil samples, 0.5 g of the dry weight equivalent from a total of 12 soil samples each was processed using the Power Soil^®^ DNA Isolation Kit (MoBio, Carlsbad, CA, United States). After extraction, the Wizard DNA Clean-Up System (Axygen Bio, United States) was employed to purify the DNA. The purity level of the DNA was at a ratio of 1.8. The DNA samples were then stored at −80°C until analysis. The copy numbers of the *nirS* and *nosZ* genes were determined using quantitative polymerase chain reaction (qPCR) with specific primer sets listed in Supplementary Table S2. For qPCR, the 20 μL reaction mixture consisted of 7.2 μL of aseptic water, 0.4 μL of each primer (10 mM), 10 μL of GoTaq^®^ qPCR Master Mix (Promega, United States), and 2 μL of template DNA. Each qPCR reaction was carried out in triplicate. Following incubation of the qPCR products at 72°C for 5 min, electrophoresis was conducted on a 2 percent agarose gel with ethidium bromide staining to facilitate detection. The known copy numbers of the target gene were used to create standard curves through a 10-fold serial dilution of plasmids containing the gene. The purified amplicons were combined in equal proportions and processed using an Illumina Miseq^®^ PE300 platform (Illumina, San Diego, United States). The amplification efficiencies and *r*^2^ values exceeded 90 and 0.99%, respectively.

### Sequencing and bioinformatics of functional genes amplicon

DNA sequencing was utilized to examine the abundance and composition of *nirS* and *nosZ* genes. The forward primers were modified with a unique 7 bp barcode sequence, and the concentration of the PCR products was measured using a TBS-380 fluorometer. Subsequently, the PCR products were diluted and subjected to paired-end sequencing on an Illumina MiSeq sequencer (Shanghai Personal Biotechnology, Co., Ltd., Shanghai, China). Additional details on the primer pairs, reaction mixtures, and thermal cycling conditions for amplifying all six genes can be found in Supplementary Table S2. Following amplification, the PCR products of all six genes were isolated from agarose gels and purified using a universal DNA Purification Kit. Quality screening of the raw sequences was performed using Quantitative Insights Into Microbial Ecology (QIIME) to ascertain any low-quality sequences (Caporaso et al., 2010). To identify chimeric assembled sequences, the Usearch tool was employed, while the FrameBot tool from the Ribosomal Database Project (RDP) was used to screen for chimeric sequences (Edgar, 2013). The FunGene Pipeline, as described by Edgar and Flyvbjerg (2015), was utilized to exclude low-quality sequences. Operational Taxonomic Units (OTUs) were defined using the CD-HIT approach within MOTHUR, with a 3% difference threshold applied to nucleotide sequences (Schloss et al., 2009). The *nirS* and *nosZ* sequences obtained spanned 31,446–56,507 bp and 34,219–59,361 bp of the valid codes, respectively. MiSeq^®^ sequencing of *nirS* obtained chimera-free reads, and average length of valid tags ranging from 223.89–224.91 bp while *nosZ* had 392.63–519.26 bp. The sequences of the *nirS* and *nosZ* genes were deposited in the NCBI Sequence Read Archive (SRA) database and can be accessed using the specific accession numbers SRR18481671 and SRR18481638, respectively.

### Statistical analysis

The data from the bulk and rhizosphere soil were subjected to one-way analysis of variance (ANOVA) using SPSS (version 22, IBM Corporation, Chicago, United States, 2013). Duncan’s multiple range tests (DMRT) were applied to determine significant differences (*p* ≤ 0.05) among the treatment means for the copy number of *nirS* and *nosZ* composition, maize grain and biomass. Alpha diversity indices, including the Shannon index, Simpson index, and Chao1 richness for *nirS* and *nosZ* genes, were determined using R software (version 3.5.3). The effects of soil physicochemical properties and *nirS* and *nosZ* Composition were evaluated using redundancy analysis (RDA) with the “vegan” package in R software. A correlation test examined the association between soil chemical properties, soil water content (SWC), maize grain and biomass. Additionally, the significant taxa in the *nirS* and *nosZ* Composition in the bulk and rhizosphere soil were identified using a co-occurrence network.

The results of four fertilization treatment samples with three replications were pooled together. The Operational Taxonomic Units (OTUs) present in each treatment replicate were chosen for network analysis. Together, Pearson correlation, Bray–Curtis, and Kullback–Leibler dissimilarities were utilized. A true co-occurrence network was defined as a statistically significant association between species when the correlation coefficient (*r*) exceeded 0.8 or was below −0.8, with a value of *p* of 0.01. Permutation and bootstrap distributions were generated with 1,000 iterations to assess the reliability of the network edges. The network was visualized using the Fruchterman–Reingold algorithm implemented in Gephi (version 0.9.2). Various topological properties of the network, including the number of nodes and edges, average clustering coefficient, average degree, average path length, closeness centrality, network centrality, and modularity, were calculated. OTUs with higher degrees and closeness centrality were identified as prospective keystone taxa. Modules are defined as the structure of networks which measures the strength of division of microbial communities (also called groups or clusters) (Berry and Widder, 2014).

Additional analysis was done using random forest modeling to ascertain the important predictors of *nirS* and *nosZ* composition and maize yield. These predictors included soil variables, and the unexpected forest package was employed (Liaw and Wiener, 2002). The significant forecasters obtained from the random forest analysis were then used to analyze the direct and indirect influences of physiochemical soil properties on biomass, network modules, *nirS* and *nosZ* communities, NUE, and maize productivity using AMOS 21.0 in SPSS (SPSS, Inc., Chicago, IL). Before modeling, the data distribution was tested for normality. A structural equation model (SEM) was employed, and the model’s fitness was assessed using the chi-square test (*χ*^2^, *p* > 0.05), root mean square error of approximation (RMSEA), and goodness-of-fit index (GFI) (Sahoo, 2019).

## Results

### Soil physiochemical parameters and yield

The analysis of variance revealed significant differences in most soil indices (TN, NO_3_^−^-N, AP, SOC, DON, and SWC) among different application rates of N fertilization in both the rhizosphere and bulk soil, except for NH_4_^+^-N. Considering the alterations in pH due to physicochemical factors, no fertilizer (N0) treatment had a higher pH than the fertilizer treatments (N1, N2, and N3). Furthermore, biotic factors such as root exudates alter the rhizosphere (8.66) pH as compared to bulk soil (8.56). The soil pH significantly varied across N fertilization rates, ranging from 8.32 to 8.66 as compared to the initial pH (8.37) of the soil before experimentation (Table 1; Supplementary Table S1). The levels of SOC, NO_3_^−^-N, AP, and DON tended to be higher in the rhizosphere compared to the bulk soil and increased with N2 and N3 treatments. Across the bulk and rhizosphere soil, the N3, N2, and N1 treatments led to an increase (13.7–27.5%; 33.5–36.1%), (12.6–21.4%; 22.7–28.9%) and (12.2–13.1%; 13.7–16.8%) compared to N0 in SOC and NO_3_^−^-N, respectively. However, N3 and N2 significantly increased TN, while the N2 treatment had higher AP levels than the N0 treatment in bulk and rhizosphere soil. The SWC was significantly higher in the bulk soil than in the rhizosphere soil across the N fertilization treatments (Table 1).

**Table 1:** Soil characteristics under different nitrogen fertilization treatments in the bulk and rhizosphere soil.

Soil	Indices	N0	N1	N2	N3
Bulk soil	pH	8.56a	8.48ab	8.40abc	8.36bc
	TN (g kg^−1^)	0.86d	0.92c	0.97bc	1.01a
	SOC (g kg^−1^)	7.55b	8.47a	8.64a	8.75a
	NO_3_^−^−N (mg kg^−1^)	18.15c	21.03b	23.49ab	27.29a
	NH_4_^+^ − N (mg kg^−1^)	16.82a	18.96a	20.31a	20.96a
	AP (mg kg^−1^)	13.56c	16.70ab	18.45a	15.91b
	DON (mg kg^−1^)	10.89c	12.42bc	14.89b	17.78a
	SWC (%)	23.11b	28.21b	32.42a	31.93a
Rhizosphere soil	pH	8.66a	8.32bc	8.44ab	8.45ab
	TN (g kg^−1^)	0.90c	1.35ab	1.93a	1.57b
	SOC (g kg^−1^)	7.73c	8.72bc	10.01b	10.66a
	NO_3_^−^−N (mg kg^−1^)	18.61c	22.36b	26.18ab	29.08a
	NH_4_^+^ − N (mg kg^−1^)	16.61a	17.82a	15.52a	17.08a
	AP (mg kg^−1^)	10.57c	17.74b	20.77a	19.16ab
	DON (mg kg^−1^)	11.38c	14.61bc	18.01b	16.81ab
	SWC (%)	13.34c	22.07a	20.46ab	16.29bc

Values are expressed as mean with letters indicating significant differences based on Duncan’s HSD test (*p* < 0.05). TN, total nitrogen; SOC, soil organic carbon; NO_3_^−^-N, nitrate nitrogen; NH_4_^+^-N, ammonia nitrogen; AP, available phosphorus; DON, dissolved organic nitrogen; SWC, soil water content. Different nitrogen fertilization rates (N0, 0 kg ha^−1^; N1, 100 kg ha^−1^; N2, 200 kg ha^−1^; N3, 300 kg ha^−1^).

The application of N fertilization treatments resulted in a significant increase (*p* < 0.05) in maize productivity compared to the no fertilizer (N0) treatment during the 2020 and 2021 cropping seasons (Supplementary Table S3). Grain yield in both seasons showed a substantial increase under N3 and N2 treatments, with (64.8, 63.2%) and (61.7, 60.8%), respectively, compared to the N0 treatment. The aboveground biomass during the same cropping seasons exhibited a similar trend, with the highest increase observed under the N3 treatment, followed by N2 and N1 treatments, with increases of (62.8, 64.9%), (58.9, 61.9%), and (48.1, 51.2%) compared to the N0 treatment, respectively (Table 2). However, there was no significant difference in grain and biomass yield between the N2 and N3 treatments (*p* > 0.05; Supplementary Table S3).

**Table 2:** Influence of fertilization on the alpha diversity indices of *nirS*- and *nosZ*-harboring denitrifiers communities at a similarity level of 97 percent in the bulk and rhizosphere soil (2021 cropping season).

Denitrifiers	Soil	Treatments	OTUs	Chao1	Shannon
*nirS*-harboring denitrifiers	Bulk soil	N0	996 ± 18.3c	349 ± 18.6b	3.58 ± 0.08c
		N1	1,487 ± 32.6b	441 ± 37.8b	3.79 ± 0.03bc
		N2	2,528 ± 31.4b	853 ± 32.5a	4.10 ± 0.03a
		N3	3,403 ± 28.3a	866 ± 24.6a	4.14 ± 0.06a
		*p*-value	<0.012	0.001	0.031
	Rhizosphere soil	N0	1963 ± 21.7c	657 ± 22.3b	5.54 ± 0.17a
		N1	2,179 ± 12.9b	1,165 ± 30.1a	5.51 ± 0.26a
		N2	4,143 ± 26.1a	1,152 ± 43.1a	5.70 ± 0.25a
		N3	3,683 ± 31.5ab	1,071 ± 33.9a	5.80 ± 0.21a
		*P*-value	<0.002	0.001	0.763
*nosZ*-harboring denitrifiers	Bulk soil	N0	2,496 ± 75.3b	1,196 ± 52.9c	4.89 ± 0.11d
		N1	3,287 ± 37.2a	1,522 ± 46.8b	5.22 ± 0.03c
		N2	2,959 ± 34.6ab	2,139 ± 59.2a	5.59 ± 0.02a
		N3	3,579 ± 19.4a	1941 ± 41.9a	5.53 ± 0.06ab
		*P*-value	<0.035	0.041	0.024
	Rhizosphere soil	N0	3,841 ± 137.2a	1848 ± 60.4c	8.27 ± 0.21b
		N1	4,736 ± 110.4a	2,385 ± 36.5b	8.57 ± 0.07ab
		N2	4,865 ± 130.5a	2,425 ± 51.6a	8.78 ± 0.16a
		N3	4,904 ± 230.2a	2,138 ± 62.5b	8.71 ± 0.07ab
		*P*-value	<0.618	0.012	0.053

Data were presented as mean with standard error. Values with the same alphabet are not statistically different, but values with different letters indicate a statistically significant difference between soil amendments on Duncan’s HSD test (*p* < 0.05). Different nitrogen fertilization rates (N0, no nitrogen fertilization; N1, nitrogen application at 100 kg ha^−1^; N2, nitrogen application at 200 kg ha^−1^; N3, nitrogen application at 300 kg ha^−1^).

The nitrogen use efficiency (NUE) exhibited a similar pattern as maize productivity. The N3 and N2 treatments resulted in a fold increase in NUE of (2.16, 1.95%) and (1.38, 1.35%) compared to the N1 treatment (Supplementary Table S3). Significant differences (*p* < 0.05) were observed among the treatments for grain yield, biomass, and NUE across the 2 years. The variation in grain yield, biomass, and NUE primarily stemmed from the year effect and the interaction between the year and treatment (Supplementary Table S3).

### Potential denitrification activity and nitrous oxide emissions

**Figure 1:**
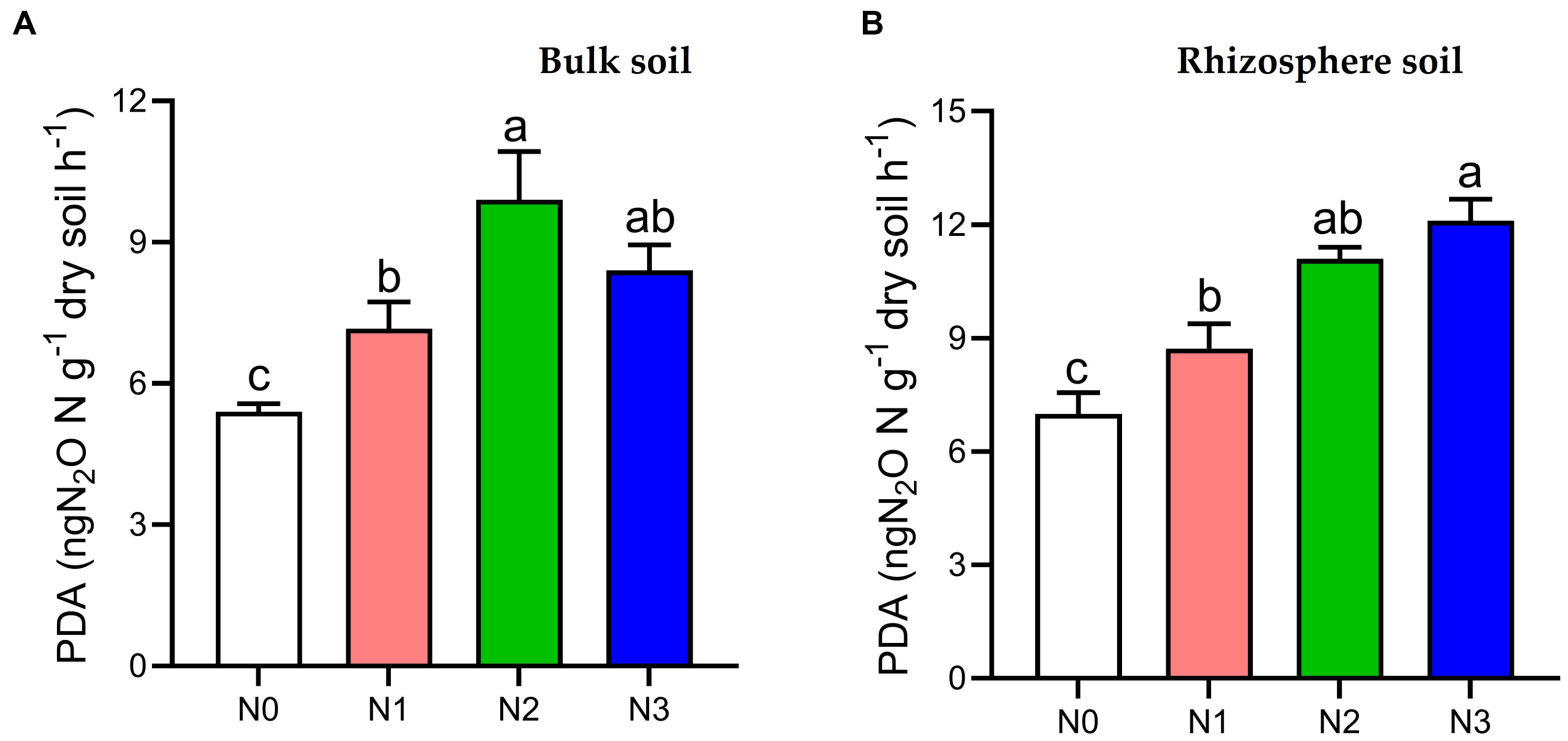
Potential denitrification activity (PDA) under different soil fertilization treatments in the **(A)** bulk and **(B)** rhizosphere soil (2021 cropping season). Bars (*n* = 3) with different lowercase letters specify significant differences based on Duncan’s HSD test (*p* < 0.05). Different nitrogen fertilization rates (N0, no nitrogen fertilization; N1, nitrogen application at 100 kg ha^−1^; N2, nitrogen application at 200 kg ha^−1^; N3, nitrogen application at 300 kg ha^−1^).

The PDA index in both bulk and rhizosphere soil showed a significant increase (*p* < 0.05) under N3 (35.7, 42.2%), N2 (45.5, 37.1%), and N1 (24.6, 19.3%) fertilization treatments compared to the N0 treatment, respectively, in the 2021 cropping seasons (Figure 1).

**Figure 2:**
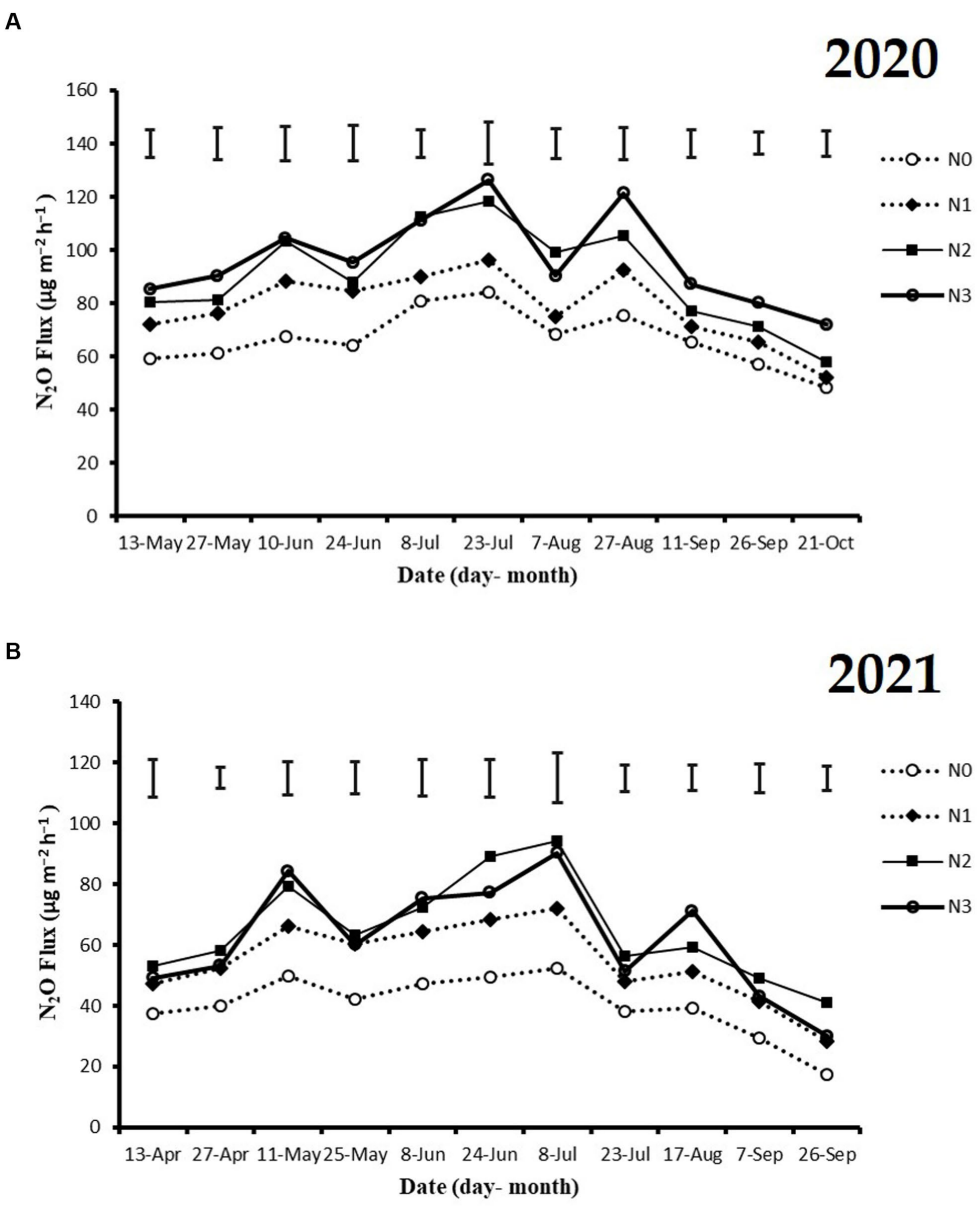
The seasonal variations of N_2_O flux emissions; **(A)** 2020 and **(B)** 2021 as influenced by fertilization treatments. The vertical bars represent the least significant difference (LSD) at *p* < 0.05. Bars (*n* = 3) with different lowercase letters indicate significant differences based on Duncan’s HSD test (*p* < 0.05). Different nitrogen fertilization rates (N0, no nitrogen fertilization; N1, nitrogen application at 100 kg ha^−1^; N2, nitrogen application at 200 kg ha^−1^; N3, nitrogen application at 300 kg ha^−1^).

**Figure 3:**
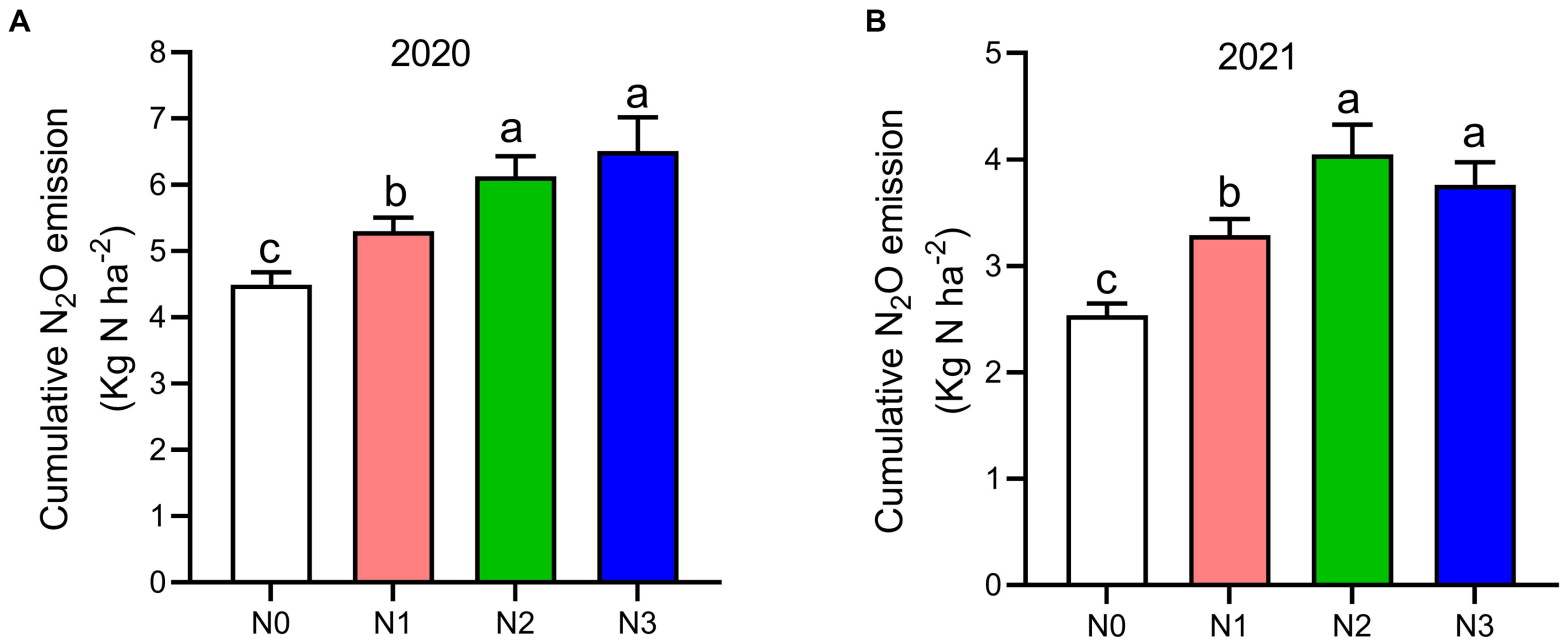
The seasonal variations of N_2_O cumulative emissions, **(A)** 2020 and **(B)** 2021 as influenced by fertilization treatments. The vertical bars represent the least significant difference (LSD) at *p* < 0.05. Bars (*n* = 3) with different lowercase letters indicate significant differences based on Duncan’s HSD test (*p* < 0.05). Different nitrogen fertilization rates (N0, no nitrogen fertilization; N1, nitrogen application at 100 kg ha^−1^; N2, nitrogen application at 200 kg ha^−1^; N3, nitrogen application at 300 kg ha^−1^).

The highest peaks of N_2_O flux emissions were observed in July, while the lowest levels were recorded in October and September across all N fertilization rates during the 2020 and 2021 cropping seasons (Figure 2A). Moreover, the release of N_2_O flux emissions was significantly higher (*p* < 0.05) under nitrogen fertilizer application (N1, N2, and N3) treatments compared to the no N fertilizer (N0) treatment during the growing seasons (Figure 2A). During the 2020 and 2021 cropping seasons, the highest N_2_O emission flux was observed under N3 and N2 treatments, measuring (126.4 and 115.7 mg m^−2^ h^−1^) and (94.2 and 86.1 mg m^−2^ h^−1^), respectively. However, the lowest N_2_O flux was recorded under N0 treatment, at 48.4 and 17.3 mg m^−2^ h^−1^, respectively (Figures 2A,B). Additionally, the cumulative N_2_O emissions in the 2020 cropping season were 32.4% higher with N3 treatment compared to N0, and in the 2021 cropping season, they significantly increased under N3, N2, and N1 treatments by 33.3, 37.3, and 22.8%, respectively, relative to N0 treatment. The emission rates ranked in the following order: N3 > N2 > N1 > N0 (*p* < 0.05; Figures 3A,B).

### Denitrification composition

**Figure 4:**
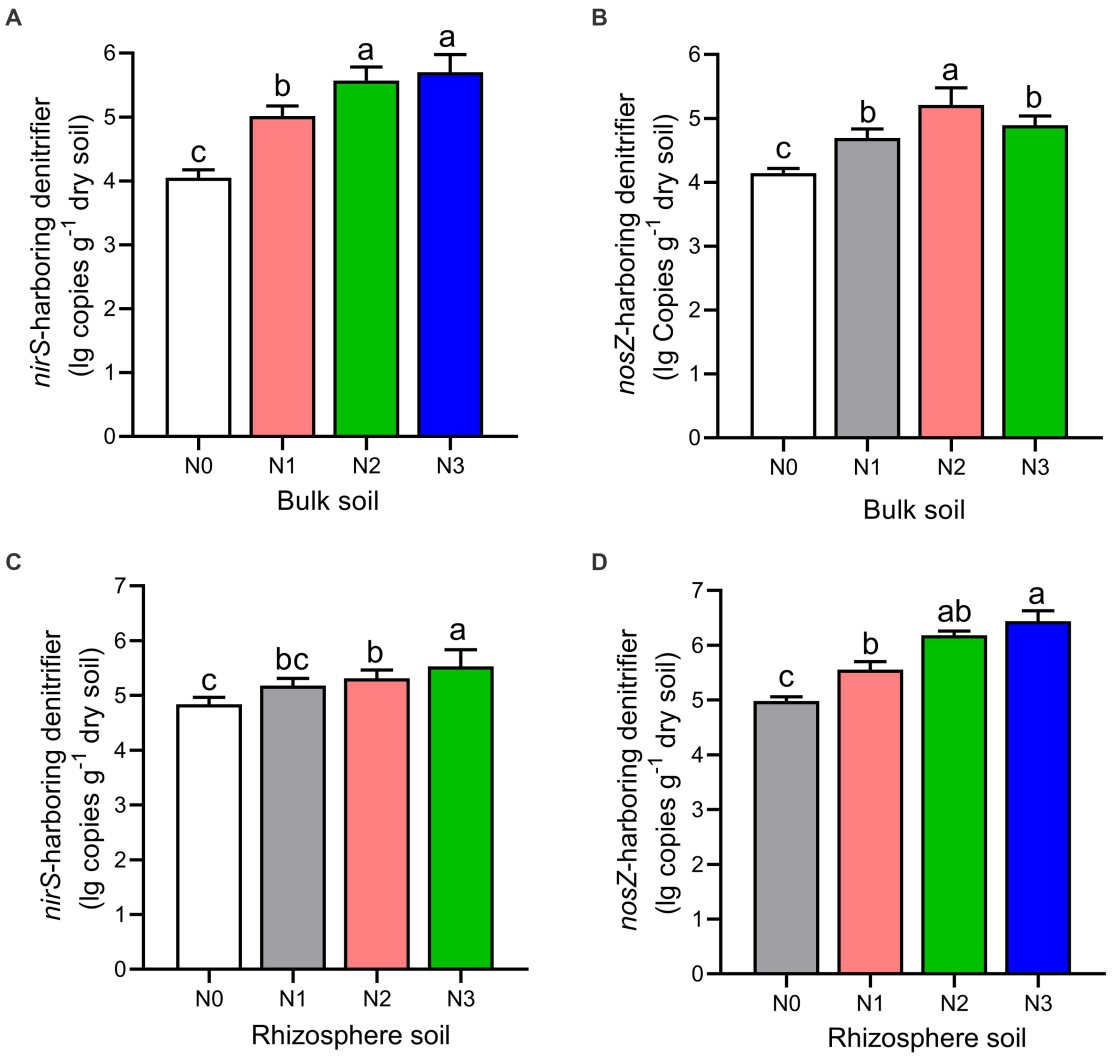
The gene copy numbers of *nirS* **(A–C)** and *nosZ* **(B–D)** genes as influenced by fertilization treatments in the bulk and rhizosphere soil (2021 cropping season). Values are mean ± standard error (*n* = 3) with different lowercase letters indicating significant differences based on Duncan’s HSD test (*p* < 0.05). Different nitrogen fertilization rates (N0, no nitrogen fertilization; N1, nitrogen application at 100 kg ha^−1^; N2, nitrogen application at 200 kg ha^−1^; N3, nitrogen application at 300 kg ha^−1^).

**Figure 5:**
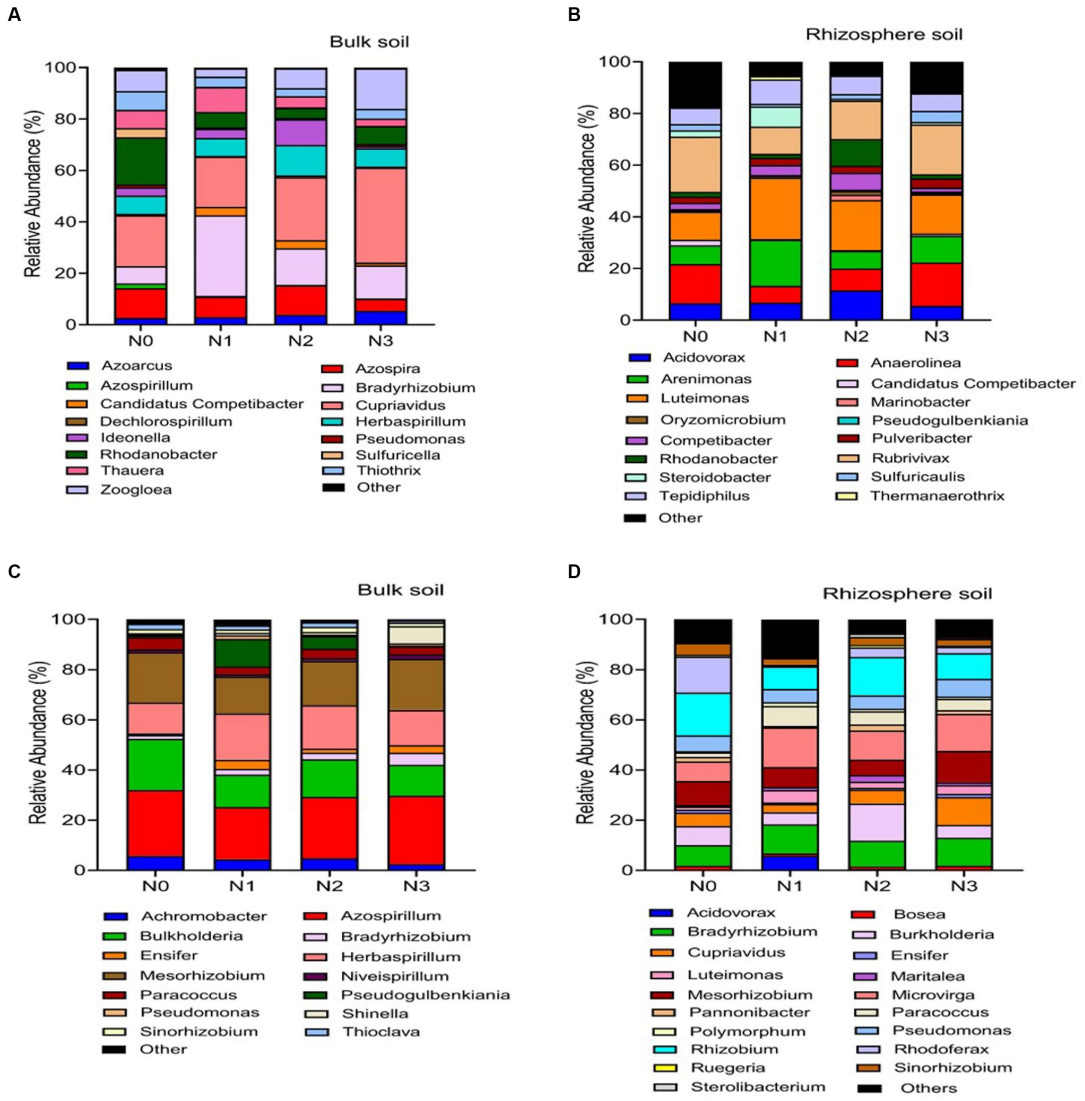
Relative abundance based on the genus level of *nirS* genes **(A–C)** and *nosZ* genes **(B–D)**. As influenced by fertilization treatments in the bulk and rhizosphere soil (2021 cropping season). Different nitrogen fertilization rates (N0, no nitrogen fertilization; N1, nitrogen application at 100 kg ha^−1^; N2, nitrogen application at 200 kg ha^−1^; N3, nitrogen application at 300 kg ha^−1^).

The rhizosphere soils exhibited higher abundance and diversity in the *nirS* and *nosZ* composition compared to the bulk soil under N fertilization rates (Figures 4A–D, 5A–D, and Table 2). Specifically, the abundance of *nirS*-harboring denitrifiers significantly increased by 20.4, 27.6, and 28.6% under N1, N2, and N3 treatments, respectively, compared to N0 treatment in the bulk soil (Figure 4A). In the rhizosphere soil, N3 and N2 treatments showed significant increases in the abundance of *nirS* and *nosZ*-harboring denitrifiers compared to N0 treatment (Figures 4C,D). Moreover, the abundance of *nosZ*-harboring denitrifiers under N2 treatment (20.5%) was significantly higher than N3 (15.4%) and N1 (11.7%) treatments in the bulk soil under N fertilization rates (Figure 4B). According to the diversity Chao1 and Shannon indices, there were substantial differences in the *nirS*- and *nosZ*-denitrifiers across N fertilization rates, with N3 and N2 treatments predominating in both bulk and rhizosphere soil (*p* < 0.05; Table 2). Compared to the bulk soil, the rhizosphere soil indicated the *nirS* denitrifier OTU diversity was considerably higher in the N0, N1, N2, and N3 treatments (*p* < 0.05; Table 2). The *nosZ*-denitrifier OTUs in the bulk soil were 30.4% (N3), 15.6% (N2), and 24.1% (N1) more diverse than in the N0 treatment, indicating a significant difference (*p* < 0.05; Table 2). However, no significant distinction existed between the N fertilization treatments in the rhizosphere soil (*p* > 0.05; Table 2).

Across the range of N fertilization rates, the genera *Cupriavidus* (22.5%), *Bradyrhizobium* (18.0%), *Rhodanobacter* (12.7%), *Azospira* (9.4%), *Herbaspirillum* (7.7%) and *Zoogloea* (7.1%) were the most prevalent *nirS*-harboring denitrifier community in the bulk soil (Figure 5A). When compared to N0 and N2 treatments, the relative abundance of genus *Cupriavidus* and *Zoogloea* significantly (*p* < 0.05) increased under N3 and N1 treatments, whereas genus *Bradyrhizobium* and *Rhodanobacter* showed the reverse trend in the *bulk* soil (Figure 5A). Whereas in the rhizosphere soil, genera *Luteimonas* (19.3%), *Rubrivivax* (16.5%), *Anaerolinea* (12.6%), *Arenimonas* (10.8%), *Acidovorax* (7.9%), *Tepidiphillus* (7.3%), and *Rhodanobacter* (5.3%) were the major *nirS*-harboring denitrifiers. Compared to N0 and N3 treatments, the relative abundance of genera *Luteimonas* was significantly (*p* < 0.05) higher under N2 and N1 treatments. In the rhizosphere soil, genera *Anaerolinea* and *Rubrivivax* established dominance under the N3 treatment (Figure 5C).

The *nosZ*-harboring denitrifiers in the bulk soil were mainly dominated by the genera *Azospirillum* (23.2%), *Mesorhizobium* (17.2%), *Burkholderia* (16.0%), and *Herbaspirillum* (14.0%) across N fertilization rates (Figure 5B). Figure 5B illustrates that genera *Azospirillum* and *Mesorhizobium* were significantly (*p* < 0.05) more abundant under N3 and N2 treatments than other N fertilizer rates, while genera *Burkholderia* showed the opposite pattern in the bulk soil. In the rhizosphere soil, the most prevalent *nosZ*-harboring denitrifiers were genera *Rhizobium* (14.2%), *Microvirga* (12.9%), *Bradyrhizobium* (10.2%), *Burkholderia* (8.8%), *Mesorhizobium* (8.3%), and *Cupriavidus* (6.3%; Figure 5D). Genera *Rhizobium* and *Burkholderia* were significantly (*p* < 0.05) more abundant in the N2 and N0 treatments compared to the N3 and N1 treatments. On the other hand, genera *Microvirga* and *Bradyrhizobium* exhibited higher abundance in soils treated with N3 compared to other N fertilizer rates (Figure 5D).

### Redundancy analysis composition

Redundancy analysis (RDA) results demonstrated that NO_3_^−^-N (19.1%), SOC (16.6%), pH (15.7%), and N_2_O emissions (13.2%) significantly (*p* < 0.05) influenced the composition of the *nirS* denitrifier composition in the bulk soil (Supplementary Table S4). The genus *Cupriavidus* exhibited a strong positive relationship with SOC and N_2_O emissions while showing a negative correlation with NO_3_^−^-N and SWC. Furthermore, NO_3_^−^-N was positively connected with the genera *Rhodanobacter*. The SWC was positively associated with the genus *Bradyrhizobium*, and SOC exhibited a negative relationship (Supplementary Figure S2A). The *nirS*-harboring denitrifier composition structure in the rhizosphere soil showed that NO_3_^−^-N (17.8%), SWC emissions (17.2%), pH (12.4%), DON (11.8%), and N_2_O emissions (10.1%) significantly increased (Supplementary Table S5, *p* < 0.05). Genera *Luteimonas* and *Rubrivivax* had a positive relationship with pH and DON and opposite relationships with SWC, NO_3_^−^-N and N_2_O emissions, respectively. However, the genera *Arenimonas* and *Acidovorax* demonstrated the reverse pattern (Supplementary Figure S2C).

The findings of RDA in the *nosZ*-harboring denitrifier composition showed that SOC (20.5%), TN (18.2%), NO_3_^−^-N (15.6%), and pH (13.5%) in the bulk soil impacted significantly (*p* < 0.05; Supplementary Table S4). Genera *Azospirillum* had a positive relationship with NO_3_^−^-N and an opposite relationship with pH, whereas *Mesorhizobium* had the reverse pattern. Genera *Burkholderia* was also positively associated with SOC (Supplementary Figure S2B). RDA study in the rhizosphere soil revealed that TN, soil pH, NO_3_^−^-N, SOC, and SWC accounted for 10.5, 10.3, 14.2, 13.4, and 12.8% of the variation in the *nosZ* denitrifying community, respectively (Supplementary Table S5; *p* < 0.05). Genera *Rhizobium* and *Cupriavidus* were identified to have a positive relationship with TN and pH. Genera *Microvirga*, on the other hand, exhibited a positive relationship with SOC (Supplementary Figure S2D).

### Co-occurrence patterns in the soil denitrification community

**Figure 6:**
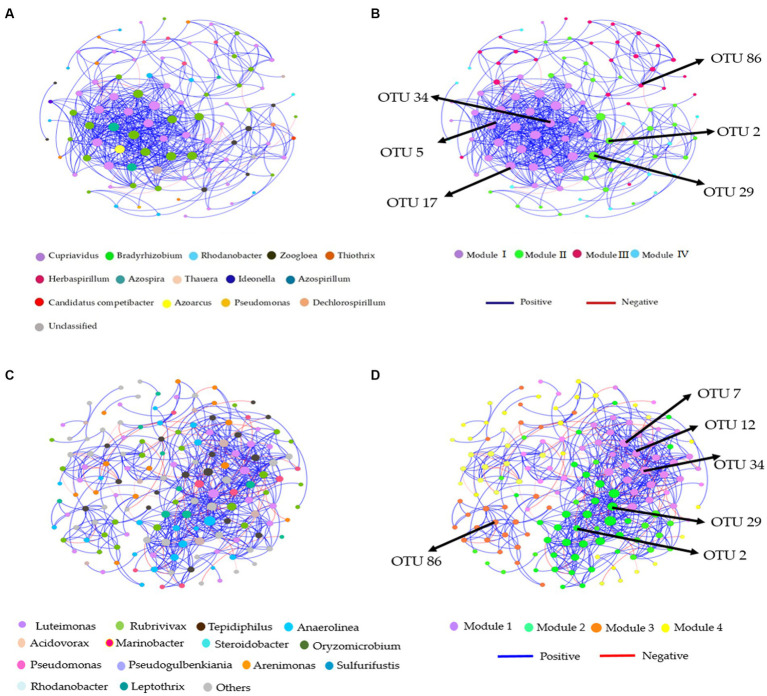
Co-occurrence network analysis of soil nitrification community at the genus level. The *nirS*-harboring network OTU taxa and modules in the **(A,B)** bulk and **(C,D)** rhizosphere soil (2021 cropping season); Modules consist of clusters that are closely interconnected nodes. The size of the OTU nodes indicates their degrees, and they are colored based on their genus-level classification. Numbers identified in the modules indicate the Keystone taxa. Blue edges represent positive, whiles red edges represent negative associations.

**Figure 7:**
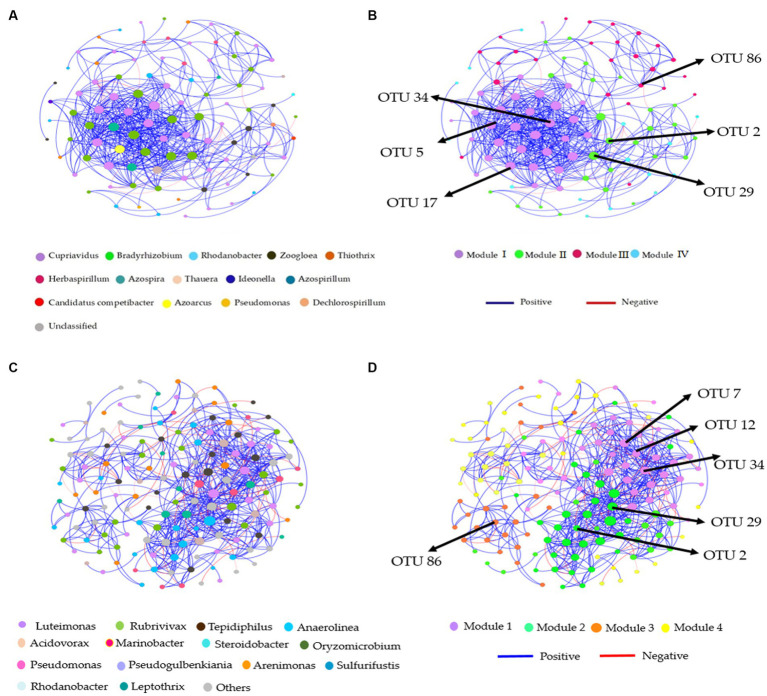
Co-occurrence network analysis of soil nitrification community at the genus level. The *nosZ*-harboring network OTU taxa and modules in the **(A,B)** bulk and **(C,D)** rhizosphere soil (2021 cropping season); Modules consist of clusters that are closely interconnected nodes. The size of the OTU nodes indicates their degrees, and they are colored based on their genus-level classification. Numbers identified in the modules indicate the Keystone taxa. Blue edges represent positive, whiles red edges represent negative associations.

Distinct modules were formed in co-occurrence networks based on treatments to analyze the soil denitrifying community. These networks were used to investigate the relationships between modules and functional groups of taxa in the bulk and rhizosphere soil (Figures 6A–D, 7A–D). Only the OTUs with a relative abundance above 0.01% in at least one replicate were included in the network. Supplementary Tables S6, S7 provide information about the module nodes and edges based on the topological characteristics of the *nirS*- and *nosZ*-harboring community in the bulk and rhizosphere soil. The *nirS*- and *nosZ*-harboring community networks in both soil types exhibited a higher number of positive associations (415 and 936 edges; 539 and 1,160 edges) compared to negative associations (8 and 34 edges; 77 and 67 edges), respectively (Supplementary Tables S5, S6). In the bulk soil, Module I exhibited a strong interconnectedness among OTUs within the *nirS*-harboring network, surpassing the relationships observed in other modules (Figure 6B). In the case of the *nosZ*-harboring network in the bulk soil, all four modules demonstrated a high level of OTU relationships, with Modules II and IV particularly prominent (Figure 7B). Additionally, in the rhizosphere soil, the *nirS*- and *nosZ*-harboring network communities displayed high OTU relationships within Modules I and II, while Modules III, IV, and V exhibited comparatively weaker relationships (Figures 6D, 7D).

The *nirS*-harboring denitrifier network in the bulk soil revealed three potential keystone taxa: genera *Cupriavidus*, *Rhodanobacter*, and *Bradyrhizobium*, indicating their critical roles within the network (Figure 6A). Similarly, the *nosZ*-harboring denitrifier network in the bulk soil indicated five keystone taxa: genera *Azospirillum*, *Mesorhizobium*, *Burkholderia*, *Ensifer*, and *Pseudomonas* emphasizing their significance within the network (Figure 7A). Furthermore, in the rhizosphere soil, the *nirS*-harboring denitrifier network was primarily influenced by the genera *Luteimonas*, *Rubrivivax*, and *Anaerolinea,* while the *nosZ*-harboring network community identified the genera *Microvirga*, *Rhizobium*, *Burkholderia*, and *Mesorhizobium* based on their network centrality and closeness centrality through the OTU modules (Figures 6C, 7C).

### Random forest modeling correlation coefficient

Using random forest modeling, our study aimed to ascertain the association between N_2_O emissions, maize yield, and potential predictors related to denitrifier communities containing *nirS* and *nosZ* genes. We examined biotic variables (such as composition, abundance, and diversity) and abiotic factors (including soil physiochemical properties). The results of the random forest modeling revealed that in the bulk soil, pH (5.2–6.1%, *p* < 0.05), SOC (8.0–8.9%, *p* < 0.01), NO_3_^−^-N (6.9–7.5%, *p* < 0.05), and DON (10.5–11.5%, *p* < 0.01) played a crucial role as indicators of abiotic variables for N_2_O emissions and maize yield (Supplementary Figures S3A,B). However, in the rhizosphere soil, the significant abiotic drivers affecting N_2_O emissions and maize productivity were pH (7.7–10.3%, *p* < 0.01), SOC (6.3–10.1%, *p* < 0.01), SWC (8.7–9.3%, *p* < 0.05), and NO_3_^−^-N (8.1–9.8%, *p* < 0.01) (Supplementary Figures S3C,D).

Additionally, the primary (biotic) factors influencing N_2_O emissions and maize yield in the bulk soil, specifically within the denitrifier communities of *nirS* and *nosZ* genes, were found to be abundance (8.7–9.6%, *p* < 0.05 and 6.7–7.7%, *p* < 0.01), composition (3.5–6.9%, *p* < 0.05 and 1.2–2.1%, *p* > 0.01) and Module I (6.1–6.4%, *p* < 0.05 and 3.4–4.3%, *p* < 0.01), respectively (Supplementary Figures S3A,B). Conversely, in the rhizosphere soil, the significant (biotic) factors affecting N_2_O emissions and maize productivity within the denitrifier communities harboring *nirS* and *nosZ* genes were identified as abundance (10.6–11.1%, *p* < 0.01 and 7.6–8.5%, *p* < 0.05), Diversity (5.5–7.4%, *p* < 0.05 and 2.3–5.0%, *p* > 0.05), Module I (4.6–6.0%, *p* < 0.05 and 2.5–5.2%, *p* > 0.05), and Module II (6.7–9.7%, *p* < 0.05; and 5.7–7.4%, *p* < 0.05), respectively (Supplementary Figures S3C,D).

### Prediction analysis between the soil denitrification communities, soil physiochemical properties, maize productivity, NUE, and N_2_O emission

**Figure 8:**
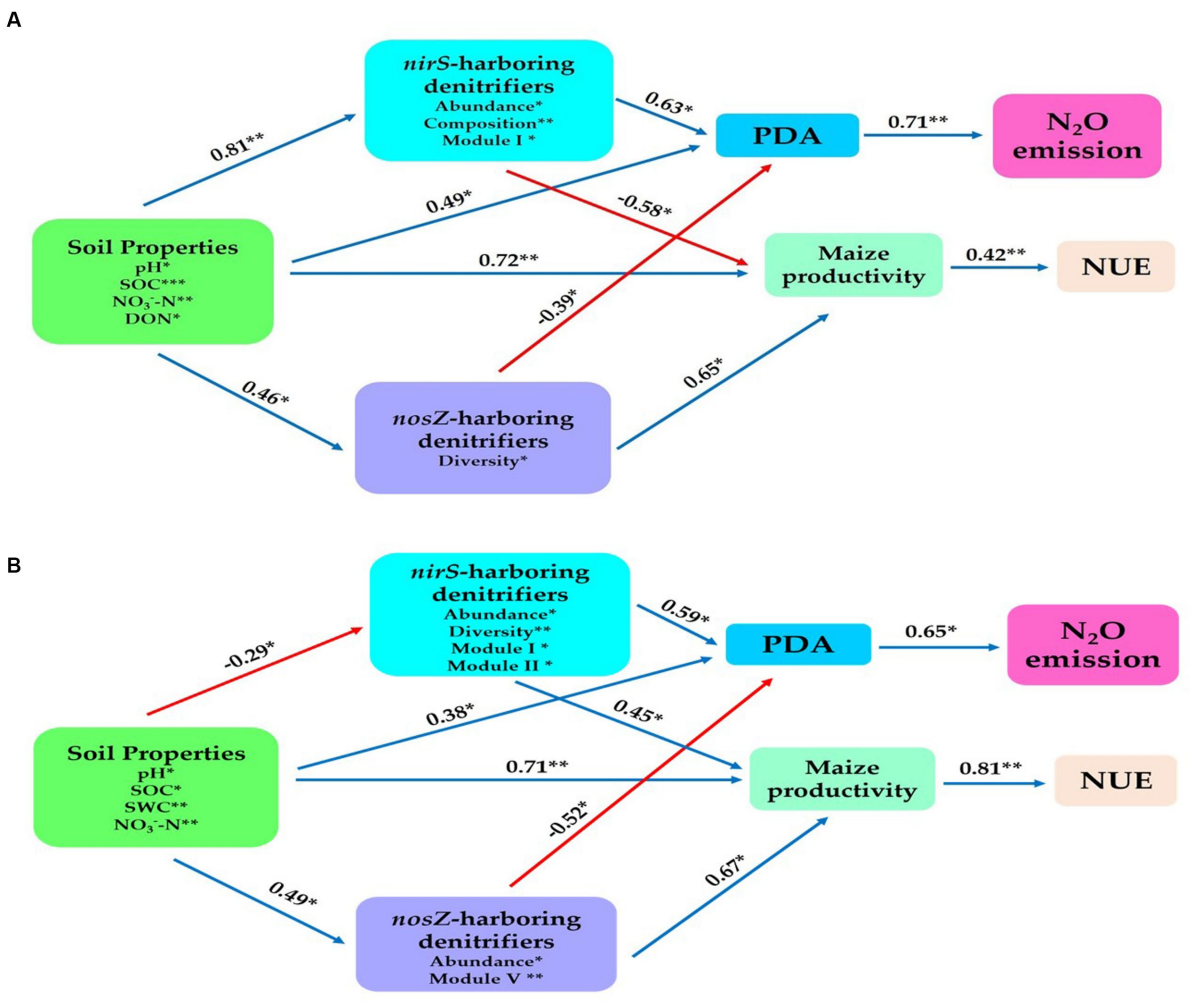
Structural equation modeling was performed to indicate the direct and indirect significant effect of soil physiochemical properties and soil nitrification community (*nirS*-, *nosZ*-harboring denitrifiers) on the N_2_O emission, maize productivity and NUE. **(A)** In the bulk soil and **(B)** rhizosphere soil (2021 cropping season). Soil properties include pH, total nitrogen (TN), soil organic carbon (SOC), available phosphorus (AP), nitrate nitrogen (NO_3_^−^-N), ammonium nitrogen (NH_4_^+^-N), and dissolved organic nitrogen (DON). The soil nitrifying community includes diversity (Shannon index), composition (first principal coordinates, PC1), and three module eigengenes in the trophic co-occurrence network. *p < 0.05 and **p < 0.01.

We established a structural equation model to establish further connections between the potential predictors, including composition, abundance, diversity, and network Modules of *nirS*- and *nosZ*-harboring denitrifiers and the abiotic drivers represented by soil properties. The aim was to examine their impact on N_2_O emissions, potential denitrification activity (PDA), maize productivity, and nitrogen use efficiency (NUE). Overall, in the bulk soil, the soil physiochemical properties (such as SOC, pH, NO_3_^−^-N, and DON) exhibited significant positive effects on the denitrifier community harboring the *nosZ* gene through abundance (*r* = 0.46, *p* < 0.05). Similarly, the *nirS*-harboring denitrifier community was influenced by abundance, composition, and Module I (*r* = 0.81, *p* < 0.01). Additionally, the soil properties positively affected maize productivity (*r* = 0.72, *p* < 0.01), and PDA (*r* = 0.49, *p* < 0.05; Figure 8A). The abundance of *nosZ* in the denitrifier community had a significant positive impact on PDA (*r* = −0.39, *p* < 0.05) and maize productivity (*r* = 0.65, *p* < 0.01). The *nirS*-harboring denitrifier community, on the contrary, had a significant positive effect on PDA (*r* = 0.63, *p* < 0.05) but a significant opposite influence on maize productivity (*r* = −0.58, *p* < 0.05) via abundance, composition, and Module I. Furthermore, a statistically significant positive relationship existed between PDA and N_2_O emissions (*r* = 0.71, *p* < 0.01). Maize yield also showed a positive association with nitrogen use efficiency (NUE) (*r* = 0.42, *p* < 0.05; Figure 8A).

The rhizosphere soil showed physiochemical characteristics of the soil (i.e. SOC, pH, NO_3_^−^-N and SWC) had significant positive impacts on the composition of the *nirS*-harboring denitrifier community through abundance, diversity, Module I and Module II (*r* = −0.29, *p* < 0.05; Figure 8B). Additionally, the *nosZ*-harboring denitrifier community was influenced by abundance and Module V (*r* = 0.49, *p* < 0.05), while maize yield (*r* = 0.71, *p* < 0.05), and PDA (*r* = 0.38, *p* < 0.05) showed significant positive relationships with the same physiochemical characteristics (Figure 8B). Furthermore, the combined effects of abundance, diversity, Module I, and Module II had a substantial positive impact on the PDA (*r* = 0.59, *p* < 0.05) and Yield (*r* = 0.45, *p* < 0.05) in the *nirS*-denitrifier community (Figure 8B). The abundance and Module V revealed a significant positive impact on maize productivity (*r* = 0.67, *p* < 0.01), while PDA had a significant negative influence (*r* = −0.52, *p* < 0.05) within the *nosZ*-harboring denitrifier community (Figure 8B). NUE and maize productivity are positively associated (*r* = 0.81, *p* < 0.01). As shown in Figure 8B, PDA had a statistically significant positive connection (*r* = 0.65, *p* < 0.05) with N_2_O emissions. The results from both the bulk and rhizosphere soil indicate that, in contrast to the *nosZ*-harboring denitrifier community, the *nirS*-harboring denitrifier community exhibited a strong positive effect with PDA activity and made a significant contribution to N_2_O emissions.

## Discussion

### Nitrogen fertilization significantly impacts the structure of soil denitrification communities

To maintain the sustainability of the microbial community composition and nitrogen-cycling processes, it is crucial to examine how nitrogen fertilization and soil physiochemical dynamics affect denitrification activities in the bulk and rhizosphere soil (Yang et al., 2018; Pang et al., 2019; Schmidt et al., 2019). Significant variations were detected in the direction of the rhizosphere soil compared to the bulk soil related to the abundance and diversity of *nirS* and *nosZ* gene copy species. This finding aligns with prior research highlighting how roots and soil pH contribute to competitive filtering, increasing biodiversity, and species richness of functional genes linked to nitrogen cycling (Han et al., 2020; He et al., 2020). The levels of nitrogen application impacted the contents of SOC, TN, and DON, which contributed to the prevalence of denitrifiers carrying the *nirS* gene, making them more notable than the *nosZ* gene. This suggests their high soil nutrient cycling and impact on the functional aspects of denitrification processes (Cui et al., 2016; Ouyang et al., 2018; Huang et al., 2020b). Substantially SOC and NO_3_^−^-N in optimal N fertilization emerged as a significant factor contributing to the elevation of denitrifier gene copies (Tao et al., 2018; Fudjoe et al., 2022). Various nitrogen fertilizer rates and environmental factors exerted distinct effects on the diversity of denitrifying communities associated with *nirS* and *nosZ*, underscoring their specialized enhancement linked to specific ecological niches that lead to heightened species richness (Yoshida et al., 2009; Jang et al., 2018; Shi et al., 2019).

Our findings indicate that alterations in the structure of denitrification communities were predominantly influenced by varying rates of nitrogen fertilization and soil properties, contributing to enhanced soil fertility. The carbon and nitrogen contents and their decomposition in the soil played a crucial role in shaping the structure of the denitrifying community (Huang et al., 2019; Chen et al., 2019a). Distinct differences were noted in the composition of denitrifying community genera between the bulk and rhizosphere soils. In our study, the bacteria genera *Cupriavidus*, *Rhodanobacter*, *Bradyrhizobium*, and *Zoogloea* were prominent in the composition of the *nirS*-harboring community in the bulk soil under the N3 and N1 fertilizer treatments. The increase in SOC and DON under different nitrogen fertilizer rate treatments underscored the significant contribution of the *nirS* denitrifying community to N_2_O emissions (Sun et al., 2017; Tao et al., 2018). *Cupriavidus* possesses metabolic pathways that utilize SOC and NO_3_^−^-N as alternative electron acceptors (Fudjoe et al., 2022). In alkaline soil, the genus *Rhodanobacter* facilitates nitrogen losses during denitrification (Ma et al., 2018). However, in the rhizosphere soil under N2 and N1 fertilizer treatments, the dominant bacteria in the *nirS*-harboring denitrifier community composition were genera *Luteimonas*, *Anaerolinea*, and *Rubrivivax*. Genus *Luteimonas* is recognized for its capacity to produce N_2_O and significant role in nitrogen biogeochemical cycles, rendering it promising for potential applications in bioremediation (Finkmann et al., 2000; Wen et al., 2017). Genus *Anaerolinea*, which belongs to the phylum *Chloroflexi*, is an oligotrophic bacterium found in alkaline soils that can decompose various carbon substrates under anaerobic conditions (Matsuura et al., 2015).

Furthermore, the *nosZ* denitrifying community stimulates the conversion of N_2_O to N_2_, reducing N_2_O emissions (Jang et al., 2018; Shi et al., 2019). The application of different rates of N fertilizers effect on the contents of DON, TN, SWC, and NO_3_^−^-N significantly increased the abundance of *nosZ* denitrifying communities in the bulk and rhizosphere soil (Ouyang et al., 2018; Han et al., 2020; Yang et al., 2021a). In the bulk soil, the main genera within the *nosZ* denitrifying communities under N2 and N3 treatments were *Mesorhizobium*, *Azospirillum*, and *Herbaspirillum*. Specifically, genera *Mesorhizobium* and *Azospirillum* were pivotal for nitrogen fixation, playing a vital role in the production of nutrient biomolecules such as organic compounds, enzymes, adenosine phosphate groups, and polynucleotides (Huang et al., 2020a; Fudjoe et al., 2022). The usage of nitrogen fertilizers resulted in the decomposition of available nutrients such as SOC and NO_3_^−^-N in the rhizosphere soil resulting in the notable prevalence of the genus *Rhizobium*, *Burkholderia*, and *Microvirga* among denitrifiers containing *nosZ*. Genus *Rhizobium* and *Microvirga* are recognized for their distinct interactions involving nitrogen fixation, antibiotic production, and phytoalexins synthesis in maize root microbiota (Sun et al., 2017; Tao et al., 2018). These findings reinforce the notion that denitrifying microorganisms residing in the rhizosphere soil play significant roles in attaining microbiome exudates and regulating N_2_O emission, consequently impacting factors related to the environment (Yang et al., 2018; Schmidt et al., 2019).

### Nitrogen fertilization influences N_2_O emissions and potential denitrification activity

Nitrogen fertilization and environmental factors are vital in increasing maize production and improving soil fertility in the semiarid Loess Plateau region. Nonetheless, this practice significantly influences the release of the emission of N_2_O from the soil, which is closely linked to plant growth and soil quality elements (Ward et al., 2017; Shi et al., 2019; Yue et al., 2022). During our research, N_2_O emissions were higher in soils treated with nitrogen fertilizer rates (N2 and N3), with the highest levels occurring in July. Moreover, plots without fertilization (N0) had comparatively lower N_2_O emissions than those with nitrogen fertilization. The study reveals that climatic conditions are crucial in determining N_2_O emission rates. The growth rate of crops, soil microbial activity, and nitrogen cycling in the soil is substantially impacted by factors such as soil temperature and moisture conditions (Peng et al., 2018; Fudjoe et al., 2022). Plastic mulch increased soil moisture and temperature (ranging from 21 to 31°C), leading to variations in drying and wetting cycles within the same period (June and July). These variations enhanced the soil water-holding capacity and temperature, improving soil aeration that affects N_2_O emissions by modifying the populations of denitrifiers (Ouyang et al., 2018; Zhang et al., 2021). Earlier studies have indicated that significant soil water stress might hinder the positive effects of temperature on nutrient accessibility to microbes (Han et al., 2020). Previous research also indicated that microorganisms regulate the allocation of SOC between cell growth and stress tolerance, thereby influencing their participation in nutrient cycling in N_2_O emissions (Cui et al., 2016; Jang et al., 2018).

Furthermore, employing various nitrogen fertilizer rates increased potential denitrification activity (PDA) in the bulk and rhizosphere soil compared to the absence of fertilizer treatment (N0). This variation can be attributed to the denitrification mechanisms that contribute to the reduction of nitrate (NO_3_^−^-N) and changes in the physiological activity of individual cells within the soil’s denitrifying communities (Yoshida et al., 2009; Yang et al., 2018). The application of N1, N2, and N3 treatments creates a favorable environment for denitrifiers primarily driven by increased quantities of organic C and N compounds, accelerating the decomposition that elevates soil nutrient turnover. Consequently, this can augment the activities of soil microbiota, thereby impacting the production of PDA and N_2_O emissions (Shi et al., 2019; Ullah et al., 2020). Our studies revealed a connection between elevated levels of nutrients such as SOC, DON, and NO_3_^−^-N and the emissions of N_2_O (Cui et al., 2016; He et al., 2020). The application of the N1 treatment had a relatively minor impact on soil N_2_O emissions compared to N3 and N2 fertilizer treatments. However, the decomposition of nitrogen fertilizer via hydrolysis led to the release of heavily oxidized nitrate substrates (NO_3_^−^-N), which subsequently caused a rise in N_2_O emissions (Ouyang et al., 2018; Fudjoe et al., 2021).

Nitrogen fertilizer stimulates crop growth, increasing root exudates and activating soil organic carbon (Chen et al., 2019b). Our study observed that the N200 and N300 treatments increased yields, NUE, and cumulative N_2_O emissions. However, there was no significant difference between these two treatments (N2 and N3), indicating that applying nitrogen fertilizer beyond 200 kg N ha^−1^ did not result in further yield increase. Our findings suggest that applying nitrogen at a 200 kg N ha^−1^ rate is advantageous in ensuring crop productivity while minimizing environmental impacts, as it achieves a high yield per unit of N_2_O emissions. Earlier research has shown that the application of 200 kg N ha^−1^ on a maize field in the semiarid Loess Plateau leads to a substantial increase in yield and nitrogen use efficiency (NUE) and also effectively limits the emission of greenhouse gases (Yue et al., 2022; Wang et al., 2023).

### Soil denitrification communities contributed to maize productivity, NUE, and N_2_O emissions

The significance of soil denitrifying communities in regulating the ecological nitrogen cycle and soil properties is essential (Ullah et al., 2020; Fudjoe et al., 2022). The utilization of N fertilizers influences traits of the plant–soil system, encompassing maize yield, nitrogen use efficiency (NUE), N_2_O emissions, and soil properties. These elements are crucial in establishing the connection between biodiversity and ecosystem function within semiarid regions (Chen et al., 2016; Liu et al., 2018a; Han et al., 2020). The presence of denitrifiers harboring the *nirS* gene positively correlated with soil nutrients (soil pH, SOC, NO_3_^−^-N and DON), PDA, and N_2_O emissions in the bulk and rhizosphere soil, indicating the differential effects. By examining the connection between maize productivity, NUE, and soil factors (abiotic and biotic), we gained insight into the primary mechanisms behind denitrification and microbial-driven nutrient cycling in both the bulk and rhizosphere soil (Yin et al., 2014; Sun et al., 2017).

In contrast, the denitrifier containing the *nosZ* gene exhibited an opposite pattern. The abundance of *nosZ* gene denitrifier in the bulk and rhizosphere soil showed a positive relationship with maize productivity and nitrogen use efficiency (NUE) and with variables such as NO_3_^−^-N, SOC, pH, SWC, and module V. The differences observed in the *nosZ*-denitrifying community could be attributed to the diffusive transport of organic substrates such as soil organic carbon and nitrogen nutrients involved actively in the cycling of nitrogen communities (Yin et al., 2014; He et al., 2020; Huang et al., 2020b). The prevalence of DON functions as a nitrate reducer leads to a significant increase in N_2_O emissions, which aligns with previous research (Yang et al., 2018; Schmidt et al., 2019). The root exudates released by plants contain a variety of soil nutrients (NO_3_^−^-N, DON, and SOC) that enrich niche distinction and functional taxonomic species of denitrifiers in the rhizosphere soil, affecting maize productivity (Chen et al., 2016; Kuang et al., 2018).

Additionally, we discovered that the network of *nirS* and *nosZ* denitrifying compositions consisted of distinct microbial modules comprising closely related species, which we identified through co-occurrence network analysis (Williams et al., 2014; Herren and McMahon, 2018). When examining the structural variances and functional processes associated with different nitrogen fertilization rates, we observed that the edges and nodes in the *nosZ* denitrifying network exhibited denser connections compared to the *nirS*-denitrifying networks in the bulk and rhizosphere soil. This discrepancy could be attributed to environmental variations and niche differentiations within the denitrifying networks (Han et al., 2020; Yang et al., 2021b). The presence of a higher proportion of positive relationships compared to negative edges in the bulk and rhizosphere soil networks indicates a mutual association that reduces competition among species within the modules of *nirS*- and *nosZ*-containing denitrifiers (Schmidt et al., 2019; He et al., 2020). The *nirS*-carrying denitrifier network exhibits a high level of centralization, facilitating efficient information transfer among discrete components of the soil microbiomes and highlighting their effective performance within the rhizosphere modules (Chen et al., 2019a; Han et al., 2020). Additionally, the *nirS*- and *nosZ*-containing denitrifiers had higher rhizosphere modularity compared to the bulk soil due to a more likely resilient links within the functional alignments and extensive network connectivity (Barberán et al., 2012; Berry and Widder, 2014; Banerjee et al., 2016). The presence of keystone species within the modules significantly impacts shaping the soil microbiome’s structure, function, and ecological steadiness (Mamet et al., 2019; Chen et al., 2019b).

Furthermore, the dynamics of nitrogen (N) fertilizer application stimulate crop growth, leading to increased root exudates from the microbiome network and improved soil physicochemical properties (Yue et al., 2022). This could contribute to the superior adaptability of biodiversity in the rhizosphere soil network compared to the bulk soil involved in nitrogen cycling (Amadou et al., 2020; Yang et al., 2021a). This distinction might be attributed to elevated soil pH, SOC, NO_3_^−^-N, maize root exudation, biomass, and litter, all contributing to enhanced soil mineralization (Schmidt et al., 2019). Therefore, our findings imply that the augmentation in N_2_O emissions could be attributed to the specific influence of soil nutrients and microbial root respiration, alongside potential competitive interactions induced by substrate from keystone taxa. These factors collectively shape the diversity of denitrification-related soil microbiomes (Ward et al., 2017; Wen et al., 2017). However, caution is necessary when making conclusions regarding the potential contribution of keystone species on denitrification communities to the overall networking system; additional stable isotope tracking is required to disentangle the underlying mechanisms further.

## Conclusion

Our research findings demonstrated that the long-term nitrogen (N) fertilization rates augmented the maize productivity, NUE and led to an increase in N_2_O emission, PDA, SOC, NO_3_^−^-N, DON, SWC, and AP but conversely decreased soil pH in the bulk and rhizosphere soil. Nitrogen fertilization had a more significant impact on the diversity and caused alterations in the composition and co-occurrence network of *nirS* and *nosZ* denitrifying communities in the bulk and rhizosphere soil. The microbial networks in the bulk and rhizosphere soil exhibited a prevalence of positive relationships rather than negative ones. This suggests a mutually beneficial relationship that reduces competition among different species of denitrifier communities. Certain important keystone species formed clusters in the microbiota ecosystem, with other genera contributing to the network resilience among the *nirS* and *nosZ* denitrifying communities. The composition and network structure of the *nosZ*-harboring denitrifier facilitated a positive impact on maize productivity but a negative effect on N_2_O emissions and PDA activities when compared to the *nirS*-harboring denitrifier, which exhibited the opposite trend. The rhizosphere soil, within the *nirS* and *nosZ* community, displayed higher microbial diversity and abundance, making it a valuable resource for promoting microbial growth and activity and improving the soil chemical environment compared to the bulk soil. Overall, the optimal N fertilization rate for a diversified soil denitrifying community was 200 kg N ha^−1^ yr.^−1^. This rate was found to be most suitable for improving spring maize yield and nitrogen use efficiency, and it could contribute to the development of sustainable nitrogen management strategies for maize production and the reduction of N_2_O emissions in the semi-arid Loess Plateau of China.

## Data availability statement

The datasets presented in this study can be found in online repositories. The names of the repository/repositories and accession number(s) can be found in the article/Supplementary material.

## Author contributions

SF: Conceptualization, Data curation, Methodology, Software, Validation, Writing – original draft, Writing – review & editing. LL: Funding acquisition, Supervision, Validation, Writing – review & editing. SA: Formal analysis, Visualization, Writing – review & editing. SS: Supervision, Writing – review & editing. JX: Project administration, Writing – review & editing. LW: Visualization, Writing – review & editing. LX: —. ZY: Visualization, Writing – review & editing.

## Funding

The author(s) declare financial support was received for the research, authorship, and/or publication of this article.

The research was supported by the Major Special Research Projects in Gansu Province (22ZD6NA009), the National Natural Science Foundation of China (32260549), the National Key R&D Program of China (2022YFD1900300) and the State Key Laboratory of Aridland Crop Science, Gansu Agricultural University (GSCS-2022-Z02).

## Conflict of interest

The authors declare that the research was conducted in the absence of any commercial or financial relationships that could be construed as a potential conflict of interest.

## Publisher’s note

All claims expressed in this article are solely those of the authors and do not necessarily represent those of their affiliated organizations, or those of the publisher, the editors and the reviewers. Any product that may be evaluated in this article, or claim that may be made by its manufacturer, is not guaranteed or endorsed by the publisher.
