# Supplementary material for: Nitrogen fertilization promoted microbial growth and N2O emissions by increasing the abundance of nirS and nosZ denitrifiers in semiarid maize field
Source: Front Microbiol. 2023 Aug 31;14:1265562. doi: 10.3389/fmicb.2023.1265562 (PMC10501401; doi:10.3389/fmicb.2023.1265562)
Supplement: Supplementary file 1 [file Data_Sheet_1.docx]

**Nitrogen fertilization induces variations in soil *nirS*- and *nosZ*-type denitrifier communities related to N_2_O emission and maize productivity in the semiarid Loess Plateau**

Setor Kwami Fudjoe^1,2^*, Lingling Li^1,2,^*, Sumera Anwar^3^, Shangli Shi^4^, Junhong Xie^1,2^, Linlin Wang^1,2^, Lihua Xie^1,2^, Zhou Yongjie^1,2^

^1^ State Key Laboratory of Aridland Crop Science, Gansu Agricultural University, Lanzhou 730070, China;

^2^College of Agronomy, Gansu Agricultural University, Lanzhou 730070, China;

^3^ Institute of Molecular Biology and Biotechnology, The University of Lahore, Lahore 54660, Pakistan;

^4^ College of Grassland Science, Gansu Agricultural University, Lanzhou 730070, China;

* **Correspondence:** [dumashiekwami@gmail.com](mailto:dumashiekwami@gmail.com)

| **Table S.1** Initial physiochemical properties in the soil before experimental setup | | | | | |
| --- | --- | --- | --- | --- | --- |
| Soil depth (cm) | BD (mg/m3) | pH | TN (g/kg) | AP (g/kg) | SOC (g/kg) |
| 0-5 | 1.19 | 8.33 | 1.05 | 0.82 | 9.91 |
| 5-10 | 1.22 | 8.32 | 1.05 | 0.74 | 8.96 |
| 0-20 | 1.28 | 8.37 | 0.94 | 0.7 | 8.89 |

Values are means (n=3). BD= Bulk density; TN=Total Nitrogen; AP= Available Phosphorus; SOC= Soil organic Carbon.

| **Target gene** | **Primer** | **Primer sequence (5’-3’)** | **Product size (bp)** | **Amplification condition** | **Reference** |
| --- | --- | --- | --- | --- | --- |
| *nirS* | *nirScd3aF nirSR3cd* | GTCAACGTCAAGGAAACCGG GACTTCGGATGCGTCTTGA | 473 | 94°C, 2.5 min  94°C for 30s, 52°C for 45s, 72°C for 8mins, 80°C for 2.5s, 32 cycles | Fudjoe et al., 2021 |
| *nosZ* | *nosZ*-F *nosZ*1622R | CGYTGTTCMTCGACAGCCAG CGSACCTTSTTGCCSTYGCG | 415 | 94°C, 5 min  94°C for 3mins, 51°C for 45s, 72°C for 55s, 80°C for 8mins, 32 cycles | Schmidt et al., 2019 |

**Table S.2 Primer pairs, reaction mixtures, and thermal cycling conditions of qPCR in this study**

*nirS* = Nitrite reductase; *nosZ* = Nitrous oxide reductase

**PCR Master Mix was purchased from Genepioneer Biotechnologies Co., Nanjing, China.**

**Table S3** Effect of different nitrogen fertilization treatments on grain yield, biomass and NUE.

| Year | Treatment | Grain yield  (kg ha^−1^) | Biomass  (kg ha^−1^) | NUE |
| --- | --- | --- | --- | --- |
|  | N0 | 4032c | 10371c | - |
|  | N1 | 8346b | 19986b | 38.5c |
| 2020 | N2 | 10543a | 25218a | 52.7b |
|  | N3 | 11461a | 27875a | 83.2a |
|  | N0 | 3416c | 7750d | - |
|  | N1 | 6293bc | 15825c | 31.9c |
| 2021 | N2 | 8724a | 20337ab | 43.6b |
|  | N3 | 9318a | 22071a | 62.1a |

Values are expressed as mean with different lowercase letters indicating significant differences based on Duncan’s HSD test (*p* < 0.05). NUE = nitrogen use efficiency. Different nitrogen fertilization rates (N0, no nitrogen fertilization; N1, nitrogen application at 100 kg ha^-1^; N2, nitrogen application at 200 kg ha^-1^; N3, nitrogen application at 300 kg ha^-1^)

**Table S3.** Analysis of variance for fertilization treatments on the year and their interaction on grain yield, biomass and harvest index.

| Source of variation | Grain yield  (kg ha^−1^) | Biomass  (kg ha^−1^) | NUE |
| --- | --- | --- | --- |
| Treatments (T) | 0.02** | 0.03** | 0.05* |
| Year (Y) | <0.01** | <0.01** | <0.02* |
| T×Y | 0.43^ns^ | 0.32^ns^ | 0.69^ns^ |

* *p*<0.05; ** *p*<0.01. *p*-value not significant (NS) at *p*>0.05 level.

**TABLE S4** Redundancy analysis output between denitrification genes and environmental variables in the bulk soil.

| Denitrifiers | Environmental variables | Explain (%) | *F* | *P* value |
| --- | --- | --- | --- | --- |
| *nirS*-harboring denitrifiers | pH | 15.7 | 2.4 | 0.018 |
|  | SWC | 11.9 | 1.8 | 0.054 |
|  | TN  N_2_O emissions | 11.7  10.5 | 1.3  1.2 | 0.062  0.050 |
|  | DON | 8.1 | 1.1 | 0.334 |
|  | AP | 7.1 | 1.0 | 0.432 |
|  | NH_4_^+^−N | 5.3 | 0.5 | 0.732 |
| *nosZ*-harboring denitrifiers | SOC | 20.5 | 3.4 | 0.002 |
|  | TN | 18.2 | 2.8 | 0.005 |
|  | NO_3_^−^−N | 15.6 | 2.4 | 0.010 |
|  | pH | 13.5 | 2.1 | 0.016 |
|  | AP | 11.2 | 1.6 | 0.102 |
|  | SWC  N_2_O emissions | 8.7  7.1 | 1.2  0.9 | 0.260  0.481 |
|  | DON | 5.4 | 0.7 | 0.660 |
|  | NH_4_^+^−N | 2.5 | 0.4 | 0.968 |

**TABLE S5** Redundancy analysis output between denitrification genes and environmental variables in the rhizosphere soil.

| Denitrifiers | Environmental variables | Explain (%) | *F* | *P* value |
| --- | --- | --- | --- | --- |
| *nirS*-harboring denitrifiers | NO_3_^-^-N | 17.8 | 2.8 | 0.063 |
|  | SWC | 17.2 | 2.7 | 0.047 |
|  | pH | 12.4 | 1.8 | 0.058 |
|  | DON  N_2_O emissions | 11.8  10.1 | 1.7  1.5 | 0.047  0.050 |
|  | SOC | 9.9 | 1.4 | 0.052 |
|  | TN | 9.0 | 1.3 | 0.235 |
|  | AP | 6.1 | 0.8 | 0.618 |
|  | NH_4_^+^−N | 2.0 | 0.3 | 0.992 |
| *nosZ*-harboring denitrifiers | NO_3_^-^-N | 14.2 | 2.2 | 0.017 |
|  | SOC | 13.4 | 2.0 | 0.024 |
|  | SWC | 12.8 | 1.9 | 0.051 |
|  | TN | 10.5 | 1.8 | 0.032 |
|  | pH | 10.3 | 1.5 | 0.134 |
|  | AP  N_2_O emissions | 8.8  7.2 | 1.2  0.9 | 0.564  0.613 |
|  | DON | 6.0 | 0.7 | 0.776 |
|  | NH_4_^+^−N | 4.2 | 0.5 | 0.904 |

**TABLE S6** Topological properties of *nirS*-, and *nosZ*-harboring denitrifier networks in the *bulk* soil.

| Denitrifiers | Module | I | II | III | IV |
| --- | --- | --- | --- | --- | --- |
| *nirS*-harboring denitrifiers | Node | 32 | 26 | 25 | 15 |
|  | Edge | 280 | 76 | 53 | 12 |
|  | Average clustering coefficient | 0.676 | 0.5145 | 0.420 | 0.223 |
|  | Average degree | 13.031 | 8.5 | 4.880 | 2.350 |
|  | Average path length | 7.196 | 5.6076 | 2.797 | 0.971 |
|  | Closeness centrality | 0.771 | 0.4018 | 0.379 | 0.653 |
|  | Network centrality | 0.506 | 0.4074 | 0.327 | 0.675 |
|  | Modularity | 0.680 | 0.429 | 0.501 | 0.212 |
| *nosZ*-harboring denitrifiers | Node | 69 | 41 | 38 | 53 |
|  | Edge | 320 | 181 | 175 | 294 |
|  | Average clustering coefficient | 0.379 | 0.381 | 0.369 | 0.416 |
|  | Average degree | 11.217 | 12.76 | 11.947 | 10.842 |
|  | Average path length | 7.495 | 8.668 | 8.011 | 7.304 |
|  | Closeness centrality | 0.489 | 0.392 | 0.544 | 0.639 |
|  | Network centrality | 0.382 | 0.407 | 0.389 | 0.382 |
|  | Modularity | 0.482 | 0.569 | 0.486 | 0.563 |

**TABLE S7** Topological properties of *nirS*- and *nosZ*-harboring denitrifier networks in the rhizosphere soil.

| Denitrifiers | Module | I | II | III | IV V |  |
| --- | --- | --- | --- | --- | --- | --- |
| *nirS*-harboring denitrifiers | Node | 49 | 45 | 24 | 36 |  |
|  | Edge | 269 | 153 | 108 | 87 |  |
|  | Average clustering coefficient | 0.648 | 0.527 | 0.391 | 0.406 |  |
|  | Average degree | 12.480 | 11.629 | 5.831 | 7.928 |  |
|  | Average path length | 8.635 | 6.134 | 3.258 | 3.161 |  |
|  | Closeness centrality | 0.572 | 0.307 | 0.584 | 0.481 |  |
|  | Network centrality | 0.431 | 0.487 | 0.531 | 0.406 |  |
|  | Modularity | 0.672 | 0.549 | 0.486 | 0.519 |  |
| *nosZ*-harboring denitrifiers | Node | 56 | 45 | 36 | 34 37 | |
|  | Edge | 247 | 405 | 260 | 189 109 | |
|  | Average clustering coefficient | 0.702 | 0.674 | 0.791 | 0.752 0.812 | |
|  | Average degree | 10.507 | 10.729 | 13.791 | 9.062 0.717 | |
|  | Average path length | 7.519 | 5.360 | 8.375 | 6.408 6.248 | |
|  | Closeness centrality | 0.653 | 0.408 | 0.716 | 0.527 0.519 | |
|  | Network centrality | 0.593 | 0.397 | 0.486 | 0.481 0.493 | |
|  | Modularity | 0.759 | 0.631 | 0.528 | 0.647 0.628 | |


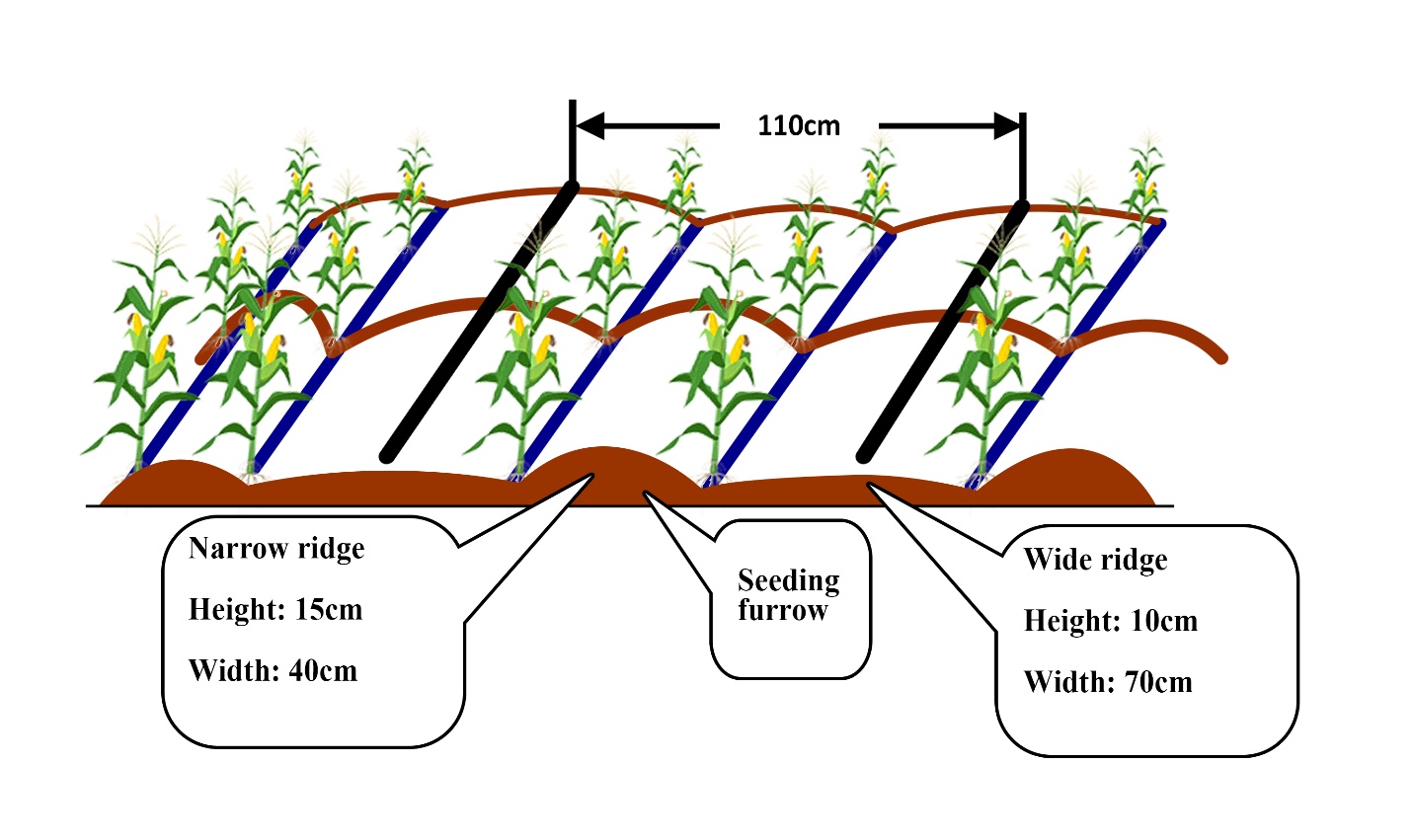


**Figure. S.1** An overview of the plot showing ridges


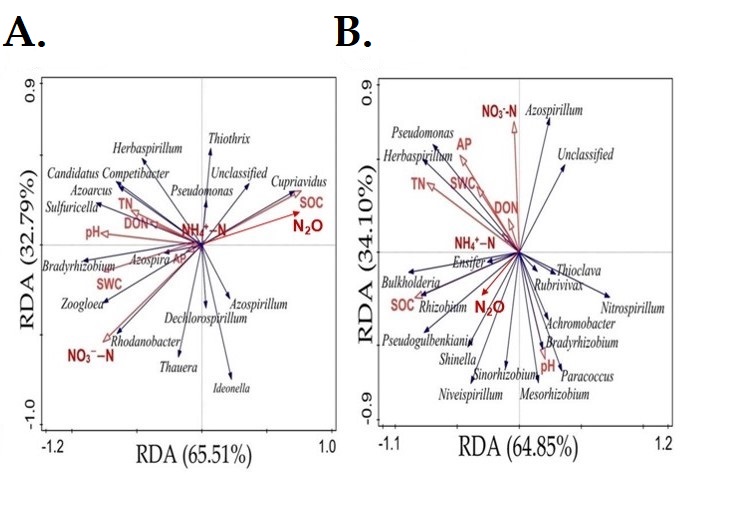


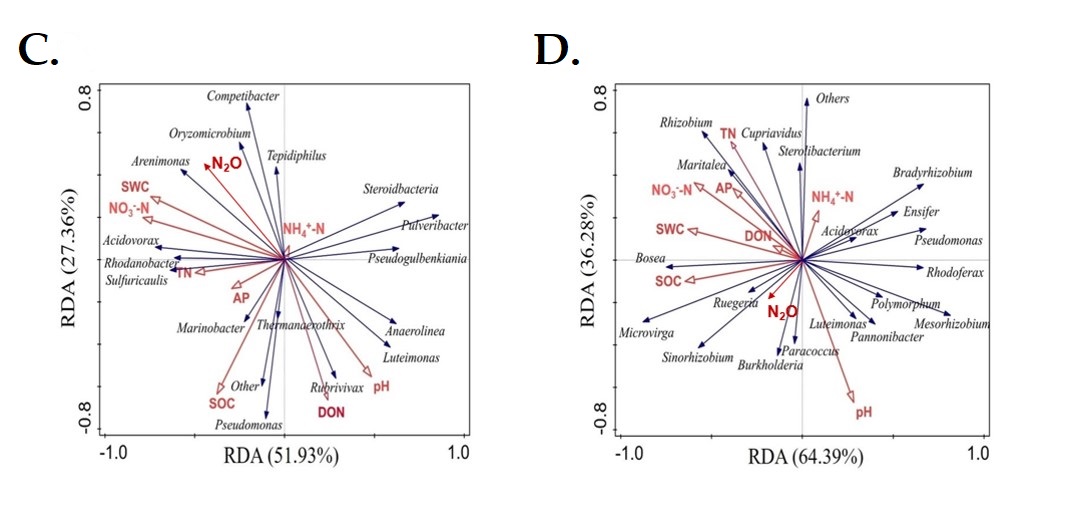


**Figure S2**. Redundancy analysis indicates the associations between soil properties and the soil denitrification community (**B-D**) ***nirS-*** and (**B-D**) ***nosZ-*** gene in the **bulk** soil and **rhizosphere** soil. TN, total nitrogen; SOC, soil organic carbon; NO_3_^−^−N, nitrate nitrogen; NH_4_^+^−N, ammonia nitrogen; AP, available phosphorus; DON, dissolved organic nitrogen; SWC, soil water content; N_2_O emission.


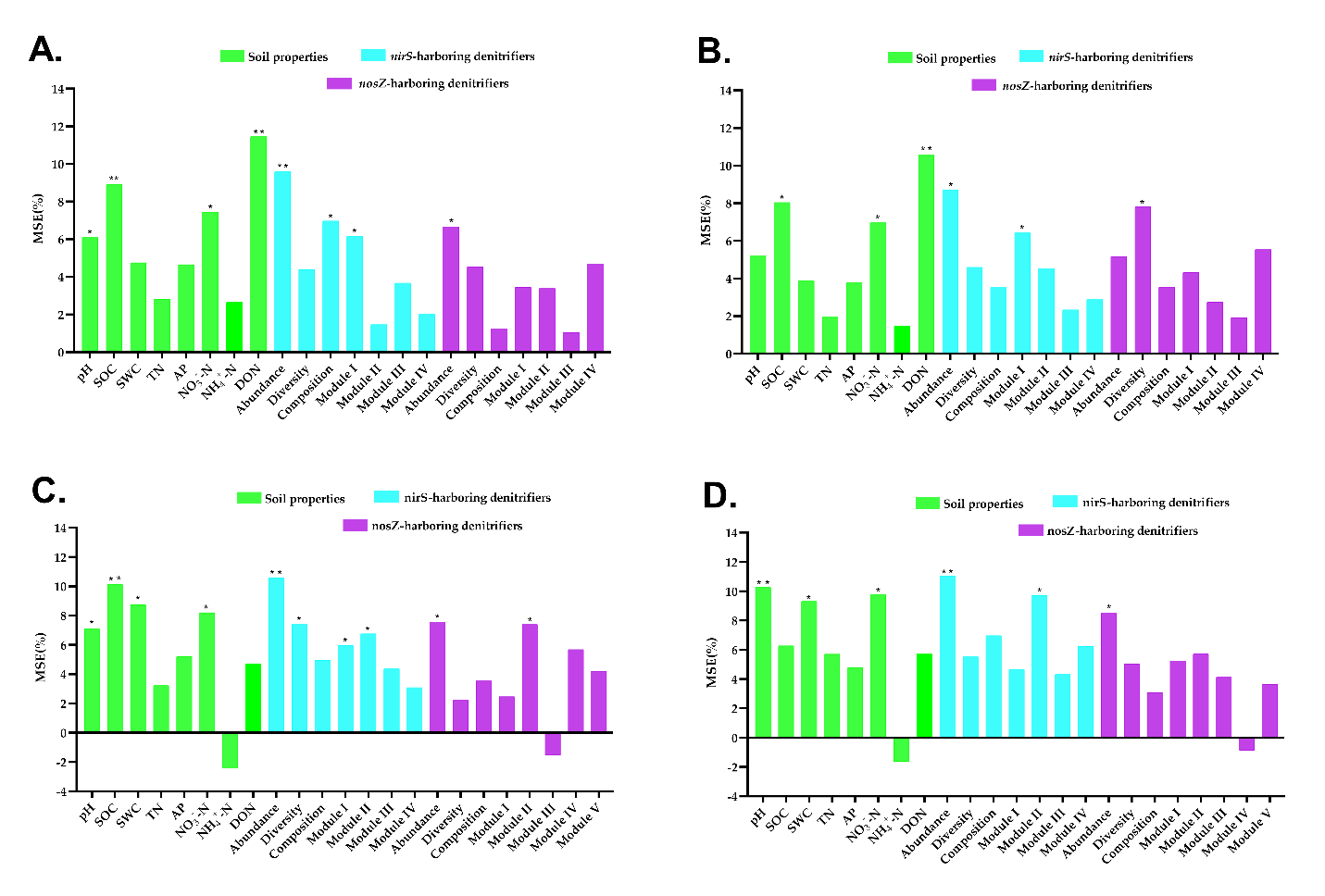


**Figure S3** Random forest modeling was performed to evaluate the contributions of soil physiochemical properties and soil nitrification community variables to **N_2_O** emission and maize productivity. (A) N_2_O emission and (B) maize productivity in the *nirS-* and *nosZ*-harboring denitrifiers in the rhizosphere soil. Random forest modeling was performed based on 12 samples (4 treatments × 3 replicates). Soil properties include pH, total nitrogen (TN), soil organic carbon (SOC), available phosphorus (AP), nitrate nitrogen (NO_3_^-^-N), ammonium nitrogen (NH_4_^+^-N), and dissolved organic nitrogen (DON). The soil nitrifying community includes diversity (Shannon index), composition (first principal coordinates, PC1), and three module eigengenes in the trophic co-occurrence network. * *p* < 0.05; ** *p* < 0.01.

**REFERENCE**

Fudjoe, S.K., Jiang, Y., Li, L., Karikari, B., Xie, J., Wang, L., Anwar, S., Wang, J., 2021. Soil Amendments Alter Ammonia-Oxidizing Archaea and Bacteria Communities in Rain-Fed Maize Field in Semi-Arid Loess Plateau. Land, 10, 10-39.

Schmidt, J. E., Kent, A. D., Brisson, V. L., Gaudin, A. C. M., 2019. Agricultural management and plant selection interactively affect rhizosphere microbial community structure and nitrogen cycling. Microbiome, 7.
